# Low IgE and absence of sensitization in non-T2 asthma: a transcriptomic and cytokine study

**DOI:** 10.3389/fimmu.2025.1711616

**Published:** 2026-01-12

**Authors:** Jyh-Hong Lee, Yu-Tsan Lin, Li-Chieh Wang, Hsin-Hui Yu, Ya-Chiao Hu, Yao-Hsu Yang, Bor-Luen Chiang

**Affiliations:** 1 Department of Pediatrics, National Taiwan University Hospital and National Taiwan University College of Medicine, Taipei, Taiwan; 2 Graduate Institute of Clinical Medicine, National Taiwan University College of Medicine, Taipei, Taiwan

**Keywords:** asthma, cytokines, immunoglobulin E (IgE), interleukin-4, RNA-seq, sensitization, type 2 inflammation

## Abstract

**Background:**

Asthma exhibits heterogeneity, including type 2 (T2-high) and non-T2 phenotypes. This study aimed to elucidate the inflammatory mechanisms that drive non-type 2 asthma, a subtype characterized by low immunoglobulin E levels and negative allergic sensitization. This context considers the complex processes of allergic sensitization.

**Methods:**

We performed gene expression analysis in non-T2 (n = 11) versus T2-high (n = 17) pediatric patients using public datasets GSE145505 for comparison (non-atopic and high-atopic datasets). We applied Ingenuity Pathway Analysis (IPA) to identify canonical pathways. We used the Database for Annotation, Visualization, and Integrated Discovery (DAVID) for functional annotation. We examined the Reactome pathway activities, focusing on differential genes related to high-affinity immunoglobulin E receptor (FcϵRI) signaling and B-cell receptor (BCR) signaling. We conducted Weighted Gene Co-expression Network Analysis (WGCNA) for a specific gene module. We also quantified the spontaneous secretion of 12 serum cytokines in an independent pediatric (non-T2, n = 50; T2-high, n = 142) and adult (non-T2, n = 111; T2-high, n = 103) asthma cohort, and we performed logistic regression to assess their associations with non-T2 asthma.

**Results:**

IPA Core Analysis predicted the inhibition of key canonical pathways in non-type 2 asthma, notably including FcϵRI signaling and BCR signaling, with the associated downregulation of calcium signaling. Gene Set Enrichment Analysis (GSEA) confirmed the significant downregulation of these and other key Reactome pathways in non-type 2 asthma, such as those related to complement activation and Fc gamma receptor-dependent phagocytosis, indicating broad suppression of pathways crucial for allergic responses. Congruently, the serum levels of interleukin-4 (IL-4) and interleukin-9 (IL-9), cytokines vital for type 2 responses, were reduced, while interleukin-2 (IL-2) levels were positively associated with non-type 2 asthma. WGCNA identified a gene module positively correlated with total immunoglobulin E that was downregulated in non-type 2 asthma; this module included interleukin-4 messenger RNA and genes like *SLC7A8* and *SIGLEC8*, suggesting impaired type 2 innate lymphoid cell function. Furthermore, transcripts for immunoglobulin heavy chain variable (IGHV), kappa variable (IGKV), and lambda variable (IGLV) gene segments were markedly downregulated. Functional annotation (DAVID) of these segments revealed enrichment for terms related to immunoglobulin production and antigen binding; their reduced function likely contributes to decreased immunoglobulin E stability and altered antigen-binding affinity, underpinning negative sensitization.

**Conclusion:**

Non-type 2 asthma represents a distinct inflammatory endotype characterized by impaired type 2 helper cell differentiation, inhibited B-cell activation and immunoglobulin E class switching, and possibly skewed immunoglobulin variable gene usage linked to altered antibody specificity. These findings suggest a multi-faceted mechanism involving broad inhibition of key sensitization and immunoglobulin E production pathways, explaining the low serum immunoglobulin E levels and the absence of allergic sensitization in non-type 2 asthma.

## 1 Introduction

Asthma is a complex, chronic inflammatory disease characterized by epithelial shedding, airway smooth muscle hypertrophy and hyperplasia, overproduction of mucus, and airway inflammation (1). The pathophysiology of asthma has been attributed to an inflammatory process that occurs predominantly in the large airways through the accumulation of eosinophils and CD4^+^ T lymphocytes in the submucosa, mucous-gland hyperplasia, thickening of the subepithelial collagen layer, submucosal matrix deposition, mast cell degranulation, and hypertrophy and hyperplasia of the airway smooth muscle (2). Chronic inflammation in asthma typically involves an increase in the number of activated CD4^+^ T cells, predominantly T helper 2 (Th2) cells. Th2 cells secrete cytokines [interleukin (IL)-4, IL-5, IL-9, and IL-13] that promote allergic inflammation and stimulate B cells to produce immunoglobulin E (IgE) (2). The clinical phenotypes of asthma have in common the propensity for acute episodes of airway obstruction and wheezing. Treatment options are not always effective and have side effects (3).

The earliest classification of asthma was extrinsic and intrinsic (4). Clinical asthma is divided into allergic (atopic) and non-allergic (non-atopic) asthma (4–6), but this is an oversimplification (2). Classifications of asthma according to phenotype and endotype have been proposed (7, 8). Based on the Th2-driven inflammatory responses, two major asthma endotypes, Th2 (T2-high) and non-Th2 (non-T2 or T2-low), have been described (9–11). T2-high asthma is characterized by upregulated type 2 immune pathways, specifically involving IL-4 and IL-13 gene expression. This subtype is associated with eosinophilic airway inflammation, airflow limitation, and higher levels of allergy, eosinophils, and bronchial hyperreactivity. This subtype is often associated with allergic conditions and positively responds to inhaled corticosteroid (ICS) treatment (12). Non-T2 or T2-low asthma includes patients with low expression of these type 2 gene markers, lacking a link to atopy and typically exhibiting a weaker short-term or refractory response to ICS. This phenotype does not respond as well to corticosteroids or T2-targeted biologics, indicating a need for alternative treatment approaches (10).

Clinical features and biomarkers that can be used to differentiate between non-T2 and T2-high asthma have been proposed, including an IgE threshold of 100 IU/mL and reactivity of both skin prick test (SPT) and radioallergosorbent test (RAST) (13). Non-T2 individuals generally have normal or low serum levels of total IgE (6, 10, 11, 14–16). Non-T2 asthma is typically defined as asthma in individuals who show no allergic sensitization, through either skin prick tests or *in vitro* specific IgE tests, to a panel of local allergens, including perennial allergens. However, the mechanism underlying the molecular endotypes of non-T2 and T2-high asthma associated with low total IgE levels and negative sensitization is unclear. The heterogeneity of non-T2 asthma hampers the identification of suitable biomarkers and therapeutic targets (16).

Allergic sensitization is the joint innate and adaptive immune response to allergens that leads to allergen-specific IgE (sIgE) synthesis (17, 18). The allergen bound to the B-cell receptor (BCR) is internalized, processed, and presented as peptides via MHC class II to T cells. This presentation triggers T-cell receptor (TCR) engagement, leading to the upregulation of CD40L on T cells, which binds to CD40 on B cells, initiating IgE class-switch recombination and IgE production in B cells (18). Plasma cells produce allergen-specific IgE that binds to FcϵRI on mast cells (MCs) and basophils. MCs are distributed throughout the body and are key cells of type I hypersensitivity reactions. FcϵRI signaling in mast cells involves a network of signaling molecules and adaptor proteins. However, during desensitization, there is an active turn-off mechanism that reduces the signaling capacity of FcϵRI. The modulation of FcϵRI signaling may modify the allergic response. Disruption of IgE–FcϵRI interactions and FcϵRI desensitization are potential therapeutic strategies (19).

We previously reported that the downregulation of immunoglobulin genes influencing FcϵRI signaling and elevated Th1 and Th17 cytokines may affect the IgE-linked sensitization associated with a non-T2 response (20). In this study, we sought to clarify the mechanisms by which immunoglobulin genes are downregulated in non-T2 asthma and to identify the molecular endotypes and canonical pathways —particularly in genes associated with FcϵRI and BCR signaling—that contribute to low IgE levels and the lack of sensitization in non-T2 asthma.

## 2 Methods

### 2.1 Asthma patients

We enrolled asthma children from the Department of Pediatrics, National Taiwan University Hospital (NTUH). Children with clinical features and characteristics suggestive of asthma were diagnosed according to the criteria of the National Asthma Education and Prevention Program Expert Panel Report 3 (EPR-3) (21) and GINA (22) (online *supplement*). The study was approved by the Institutional Review Board and Research Ethics Committee of the National Taiwan University Hospital (202206025RINC) and adhered to the tenets of good clinical practice and to the principles of the Declaration of Helsinki. Informed consent was obtained from the participants and/or their legal guardians.

### 2.2 Serum levels of total and allergen-specific IgE

Total IgE levels were measured using the CAP FEIA System (Pharmacia, Uppsala, Sweden) according to the manufacturer’s instructions. The data were calibrated using the World Health Organization standard within the range of 2–5,000 IU/mL. Allergen sensitization was determined using the MAST Optigen test (Hitachi Chemical Diagnostics, Mountain View, CA, USA).

Children were defined as having T2-high asthma when they had a positive allergen-specific IgE level (positive sensitization; allergen-specific IgE > 0.35 IU/mL) in accordance with clinical symptoms (history of cough, recurrent wheezing, recurrent difficulty breathing, and recurrent chest tightness). In contrast, children were defined as having non-T2 asthma when they had no personal or family history of allergic symptoms and in whom allergic sensitization was not identified (via skin prick or *in vitro* allergen-specific IgE tests with allergen-specific IgE < 0.35 IU/mL) to a panel of local allergens, including dust mites, dander, feathers, molds, grasses/trees, foods, cockroach mix, and latex (20). We enrolled a discovery cohort comprising 11 non-T2 and 17 T2-high asthma patients.

### 2.3 mRNA expression dataset

mRNA data from peripheral whole blood of non-T2 asthma *vs*. T2-high asthma using bulk RNA-seq (study dataset) have been described previously (20). We used the publicly available GSE145505 dataset of mRNA expression data (log_2_FC and *p*-value) was downloaded from the Gene Expression Omnibus (GEO) (BioProject PRJNA607333; series accession GSE145505) (23). Using Ingenuity Pathway Analysis (IPA), we compared the seven gene datasets in GSE145505 with those from our study dataset to identify canonical pathways.

### 2.4 Gene Set Enrichment Analysis

Gene Set Enrichment Analysis (GSEA) is a computational method used in bioinformatics to determine whether a predefined set of genes shows statistically significant, concordant differences between two biological states (e.g., non-T2 *vs*. T2-high) (24, 25). We utilized the C2 collection of curated gene sets from the Molecular Signatures Database (MSigDB) (https://www.gsea-msigdb.org/gsea/msigdb/collections.jsp) (online *supplement*).

### 2.5 Determination of serum levels of cytokines

From an independent cohort of asthma patients made up of both children (non-T2 asthma, n = 50; T2-high asthma, n = 142) and adults (non-T2 asthma, n = 111; T2-high asthma, n = 103), we collected serum samples to validate whether the cytokine levels are related to children and adults with non-T2 asthma. We conducted simultaneous quantification of IL-2, 4, 5, 6, 9, 10, 13, 17A, 17F, 22, IFN-γ, and TNF-α. We measured these cytokines in serum samples using the Human Th Cytokine Panel (12-plex) with V-bottom Plate V02 (Cat. No. 741028; BioLegend Inc., San Diego, California (CA)). We conducted logistic regression to assess the associations between non-T2 asthma and the serum levels of IgE and 12 cytokines.

We performed univariate logistic regression to evaluate the association of each independent variable, including IgE and cytokines, with non-T2 asthma individually, without adjusting for other variables. Then, we conducted multivariate logistic regression to assess the combined effects of multiple predictors on non-T2 asthma. In one model, we included both the IgE level and the levels of 12 cytokines as continuous variables. In another model, we included IgE as a categorical variable (threshold 100 IU/mL) with the levels of 12 cytokines as continuous variables. We set statistical significance at *p* < 0.05 and performed the analyses using the SAS software (v. 9.4, SAS Institute, Cary, NC, USA).

### 2.6 Weighted Gene Co-Expression Network Analysis

Weighted Gene Co-Expression Network Analysis (WGCNA) examines co-expression patterns, revealing functional networks and reducing reliance on arbitrary gene expression thresholds. WGCNA is a systems-level approach for identifying gene modules, offering insight into complex biological processes (26). The R package WGCNA (v. 1.66) was used for co-expression analysis (online *supplement*).

### 2.7 Ingenuity Pathway Analysis

We performed Core Analysis with IPA (Qiagen, Redwood City, CA, USA) to identify related biological pathways, upstream regulators, and molecular interactions. We analyzed Reactome pathways to corroborate the findings of the canonical pathway analysis (27). The Reactome Pathway Browser provided an interactive schematic (https://reactome.org/). We performed a comparison analysis of IPA for all datasets (ours and seven GSE145505 datasets). We accessed the canonical pathways of all eight datasets and calculated the z-scores determined via pathway activity analysis. We accessed the Reactome pathway using the IPA platform, where we used purple to highlight genes or nodes included in the dataset analysis results (online *supplement*).

### 2.8 Functional annotation and pathway enrichment analysis

To explore the potential biological functions of genes within the selected seven Reactome pathways, we conducted a pathway enrichment analysis using the Database for Annotation, Visualization, and Integrated Discovery (DAVID) (https://david.ncifcrf.gov/). We examined functional annotation charts for all genes included in the selected Reactome pathways.

## 3 Results

### 3.1 Selection and definition of the mRNA dataset

To understand the transcriptional differences between non-T2 and T2-high asthma, we conducted a DESeq2 analysis comparing gene expression between the non-T2 (n = 11) *vs*. T2-high (n = 17) patient groups (**Table 1**). Initially, we analyzed 44,076 genes. We identified no outliers, with a pre-filter median read count of 20,003 genes. We named this the study dataset. Based on providing a spectrum of severity and phenotypes of GSE145505 datasets, we ranked these seven datasets from the lowest to highest atopy (**Table 2**). We named the GSE145505.GPL16791.DESeq2R.test27 dataset as the non-atopic dataset, and the GSE145505.GPL16791.DESeq2R.test22 dataset as the high-atopic dataset. Both datasets had pre-filter median read counts of 38,525 genes.

**Table 1:** Patients characteristics of the non-T2 (n = 11) *vs*. T2-high (n = 17) patient groups.

Characteristic	Non-T2 asthma (n = 11)	T2-high asthma (n = 17)
Age at enrollment: years, mean ± SD (range)	10.72 ± 5.24 (3.50–21.33)	9.86 ± 5.84 (3.00–21.58)
F:M (ratio)	6:5	8:9
Total serum IgE (IU/mL; mean ± SD) (range)	41.12 ± 32.57 (4.03–100)	405.84 ± 252.12 (54.3–960)
Peripheral blood eosinophil (%; mean ± SD) (range)	2.01 ± 1.78 (0.10–4.80)	4.14 ± 3.22 (0.20–12.60)
Sensitization rate (%)		
Dust mites	0	100.00
Dander	0	55.60
Molds	0	22.20
Grass	0	22.20
Foods	0	5.60
Cockroach	0	0
Latex	0	0

**Table 2:** Comparison analysis based on activity z-score trend for nine top-ranked pathways across the eight datasets (present study and GSE145505).

Canonical pathways	Study dataset	Non-atopy (non-atopic dataset)	Transient wheeze, low atopy	Low wheeze, low atopy	Low wheeze, high atopy	Medium wheeze, low atopy	High wheeze, high atopy, low lung function	High wheeze, high atopy (high-atopic dataset)
Cell surface interactions at the vascular wall	−5.96	−4.025	−1.961	−1.723	−2.722	1.606	0.557	1.508
Immunoregulatory interactions between a lymphoid and a non-lymphoid cell	−7	−6.119	−3.286	−3	−3.841	3.569	0	−1.033
Communication between innate and adaptive immune cells	−8.718	−4.714	0.302	0.742	−2.832	1.961	−0.539	1.896
TRIM21 intracellular antibody signaling pathway	−8.488	−3.244	0.164	2.692	−0.686	1.279	−0.577	3.795
Complement cascade	−6.505	−3.124	−0.557	1.043	−1.89	2.183	−0.6	3.286
Binding and uptake of ligands by scavenger receptors	−7.071	−2.596	−1.89	0.146	−1.569	0.277	−1.569	3.528
Fc gamma receptor (FCGR) dependent phagocytosis	−6.928	−3.286	−0.655	1.067	−1.225	1.155	−0.943	2.785
Signaling by the B-cell receptor (BCR)	−6.091	−2.6	−0.426	1.768	−0.471	1.265	−0.535	4.491
Fc epsilon receptor (FcϵRI) signaling	−6.856	−3.024	−1.091	0.302	−1.279	0.707	−1	2.785

Note. a) Clinical phenotype (wheezing and atopy) classification was derived from Altman et al. (23) and GSE145505 data in OmicSoft. b) Numbers represent pathway activity z-scores calculated in Core Analysis of Ingenuity Pathway Analysis (IPA). An activity z-score is a statistical measure used to predict the activation state of biological functions or pathways based on experimental data. A positive z-score (≥2) greater than or equal to 2 predicts that the function or pathway is activated. A negative z-score (≤−2) predicts that the function or pathway is inhibited. A z-score close to 0 indicates no predicted change in activity.

### 3.2 Inhibited reactome pathways in non-T2 asthma identified by IPA core analysis

**Figure 1:**
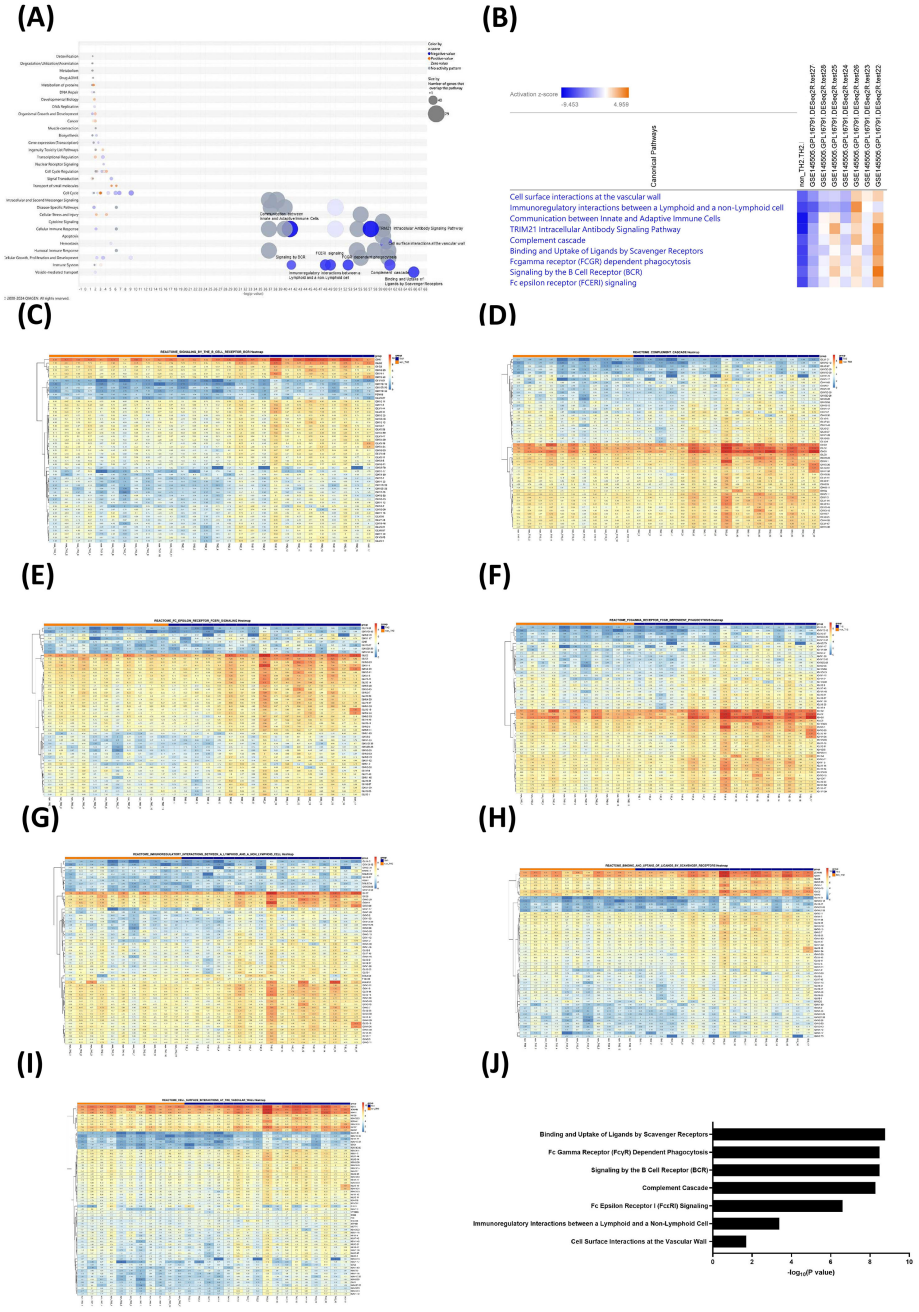
Significance of canonical pathway activity and Reactome pathway enrichment distinguishing non-T2 from T2-high Asthma. **(A)** Bubble chart illustrates the canonical pathway enrichment analysis of gene dataset derived from RNA-seq of peripheral blood from non-T2 asthma *vs*. T2-high asthma (study dataset) using Core Analysis of Ingenuity Pathway Analysis (IPA; Qiagen). Each row represents a biological pathway categorized by function, while the x-axis denotes the −log (*p*-value), indicating the statistical significance of enrichment. Bubble size corresponds to the number of genes overlapping with the pathway, and color indicates the z-score, reflecting pathway activation (orange for positive z-scores) or inhibition (blue for negative z-scores). Gray bubbles signify pathways without a discernible activity pattern. Significant enrichment of pathways is specifically labeled, particularly among highly suppressed pathways (rightmost blue bubbles). **(B)** Comparative analysis of canonical pathway activity across eight gene datasets (study dataset plus seven gene datasets from GSE145505). The heatmap presents the activation z-scores of key canonical pathways across eight datasets. The color gradient represents pathway activation, ranging from negative z-scores (blue, indicating inhibition) to positive z-scores (orange, indicating activation). The analysis highlights key pathways that show differential activation patterns and exhibit significant variability in activity across datasets. There appears to be a progression from lower (negative) z-scores in the left side (non-T2 or non-atopic) datasets to higher (positive) z-scores in the right side (T2-high or high-atopic) datasets. Signaling by the B-cell receptor (BCR)B-cell receptor (BCR) **(C)**, complement cascade **(D)**, high-affinity immunoglobulin E receptor (FcϵRI) signaling **(E)**, FcγR-dependent phagocytosis **(F)**, immunoregulatory interactions between a lymphoid cell and a non-lymphoid cell **(G)**, binding and uptake of ligands by scavenger receptors **(H)**, and cell surface interactions at the vascular wall **(I)**. Gene Set Enrichment Analysis (GSEA) Reactome gene sets from non-T2 asthma *vs*. T2-high asthma groups **(J)**. Rows represent individual genes associated with pre-defined GSEA gene set, while columns represent samples within each asthma group. The hierarchical clustering on the left side indicates that the gene expression profiles segregate distinctly between the two groups. Certain distinctive gene clusters (in red rectangle) appear to have the highest variability and differential expression across groups. These clusters may represent key drivers of the pathway’s behavior in each GSEA gene set. Statistical significance was determined with a false discovery rate (FDR) <0.25, with ranked gene lists generated from differential expression analysis. Statistical significance of enrichment [−log (*p*-value)] for GSEA Reactome pathways distinguishing non-T2 asthma from T2-high asthma groups.

We performed canonical pathway analysis to investigate the biological processes distinguishing non-T2 from T2-high asthma. Core Analysis of the study dataset revealed that binding and uptake of ligands by scavenger receptors, complement cascade, cell surface interactions at the vascular wall, TRIM21 intracellular antibody signaling pathway, Fcγ receptor (FCGR)-dependent phagocytosis, Fcϵ receptor (FcϵRI) signaling, immunoregulatory interactions between a lymphoid cell and a non-lymphoid cell, signaling by the BCR, and communication between innate and adaptive immune cells were the top-ranked inhibited canonical pathways (**Figure 1A**). Seven were Reactome pathways (**Table 3**); the *TRIM21 intracellular antibody signaling pathway* and *communication between innate and adaptive immune cells* were not.

**Table 3:** Seven reactome pathways revealed by GSEA.

ID	Gene set size	Normalized enrichment score (NES)	False discovery rate (FDR)	*p*-Value
REACTOME_BINDING_AND_UPTAKE_OF_LIGANDS_BY_SCAVENGER_RECEPTORS	80	−3.10892	0.025274083	0.002237136
REACTOME_FCGAMMA_RECEPTOR_FCGR_DEPENDENT_PHAGOCYTOSIS	138	−3.10805	0.025274083	0.002212389
REACTOME_SIGNALING_BY_THE_B_CELL_RECEPTOR_BCR	163	−2.99441	0.025274083	0.002325581
REACTOME_COMPLEMENT_CASCADE	89	−2.9776	0.025676721	0.002336449
REACTOME_FC_EPSILON_RECEPTOR_FCERI_SIGNALING	182	−2.89675	0.025274083	0.002320186
REACTOME_IMMUNOREGULATORY_INTERACTIONS_BETWEEN_A_LYMPHOID_AND_A_NON_LYMPHOID_CELL	172	−2.86861	0.025274083	0.002314815
REACTOME_CELL_SURFACE_INTERACTIONS_AT_THE_VASCULAR_WALL	166	−2.58274	0.025274083	0.002298851

GSEA, Gene Set Enrichment Analysis.

To explore a spectrum of atopy levels, we also performed Core Analysis of the seven GSE145505 datasets. We used hierarchical clustering in the comparison analysis of IPA to group canonical pathways of all eight datasets. The top-ranked canonical pathway identified in the study group was common to all seven GSE145505 datasets (**Figure 1B**). The activation status (z-score) of these identified pathways exhibited a dose-dependent effect as the degree of atopy progressed from non-atopic to high-atopic (**Table 2**). The study and non-atopic datasets exhibited predominantly negative z-scores, suggesting widespread pathway inhibition. The middle datasets displayed a mix of activation and inhibition, indicative of a transitional state. The high-atopic datasets showed strong positive z-scores, implying overall pathway activation, with their activity increasing in a dose-dependent manner as atopy increases in severity. These results support the idea that these pathways are linked to the atopic/T2-high phenotype and are suppressed in non-atopic/non-T2 asthma, suggesting that these pathways play a concerted role in determining the degree of atopy.

### 3.3 GSEA reveals distinct pathway enrichment in non-T2 *vs*. T2-high asthma

To distinguish between non-T2 and T2-high asthma, GSEA of 11 non-T2 and 17 T2-high patients identified the same seven Reactome pathways identified by IPA (**Table 3**). The heatmaps reveal that each Reactome pathway displayed a unique expression profile across the two patient groups—non-T2 *vs*. T2-high—indicating distinct transcriptional states and underlying immune mechanisms for each asthma subtype (**Figures 1C–I**). The non-Th2-enriched pathways predominantly involve innate immune mechanisms, including complement activation, Fcγ receptor-mediated phagocytosis, and scavenger-receptor pathways (**Figure 1J**).

### 3.4 Lower Th2 cytokine levels and inverse correlation with total IgE levels in non-T2 asthma confirmed in independent cohort

To validate the observed transcriptional differences at the protein level, the serum levels of cytokines secreted by Th1, Th2, Th9, Th17, and Th22 cells were determined in the independent cohort of a total of 406 subjects stratified into pediatric and adult (n = 214) cohorts based on age. Within each cohort, patients were further classified into T2-high (n = 192) and non-T2 (n = 161) asthma phenotypes based on their sensitization status. In the pediatric group, 142 patients (74.0%) were classified as T2-high and 50 patients (26.0%) as non-T2. The mean age was comparable between the two groups, with no statistically significant difference observed (T2-high: 9.6 ± 4.4 years *vs*. non-T2: 8.9 ± 4.1 years; *p* = 0.34). As expected, total serum IgE levels were markedly elevated in the T2-high group compared to the non-T2 group (650.3 ± 575.0 *vs*. 85.6 ± 113.0 IU/mL, respectively), representing a statistically significant difference (*p* < 0.001). In the adult group, 103 patients (48.1%) were classified as T2-high and 111 patients (51.9%) as non-T2. Unlike the pediatric cohort, there was a significant difference in age between the phenotypes; the T2-high adults were significantly younger (36.1 ± 10.9 years) compared to the non-T2 adults (45.0 ± 11.9 years; *p* < 0.001). Consistent with the pediatric findings, the adult T2-high group exhibited significantly higher total serum IgE levels compared to the non-T2 group (483.3 ± 492.5 *vs*. 54.0 ± 74.8 IU/mL; *p* < 0.001).

**Figure 2:**
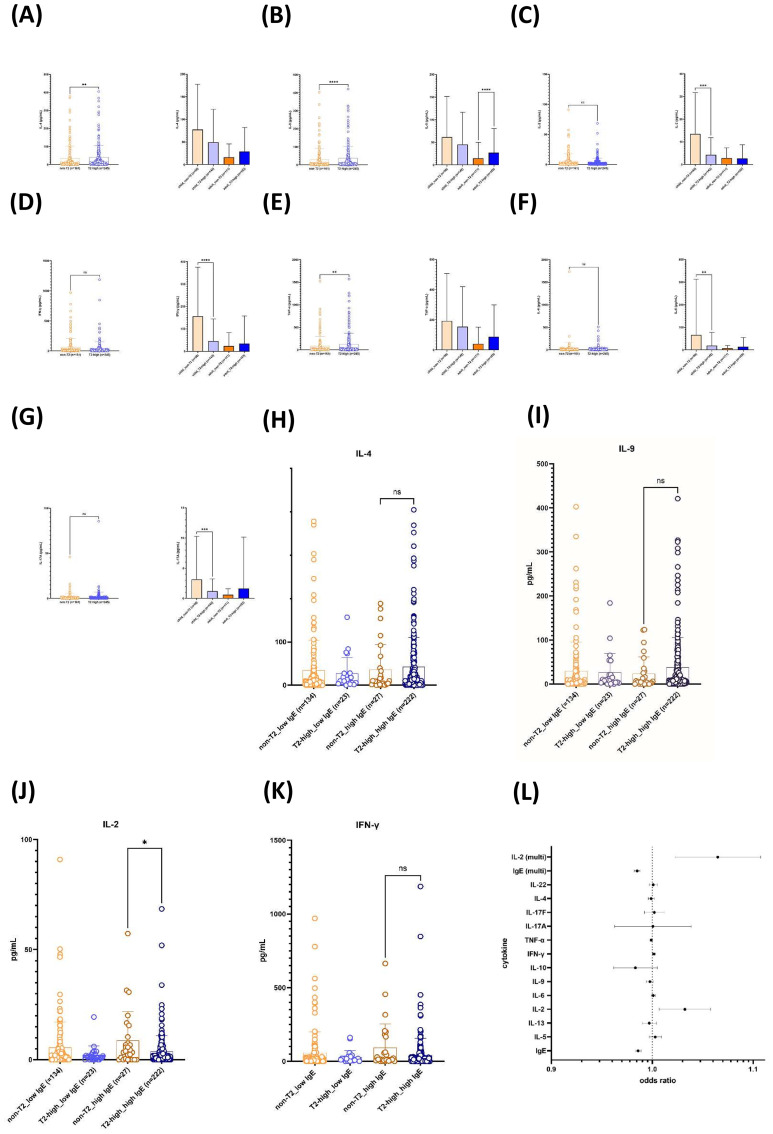
Serum cytokine profiles differentiate T2-high from non-T2 asthma phenotypes across pediatric and adult cohorts. **(A–G)** Serum concentrations of key inflammatory cytokines were quantified by multiplex immunoassay in pediatric (*n* = 192) and adult (*n* = 214) asthma cohorts. Patients were stratified into T2-high and non-T2 endotypes based on the Methods section. **(A, B)** Type 2 cytokines **(A)** IL-4 and **(B)** IL-9 were significantly elevated in the T2-high groups compared to non-T2 counterparts in both age cohorts, confirming the T2-high stratification. **(C–E)** Th1-associated cytokines **(C)** IL-2, **(D)** IFN-γ, and **(E)** TNF-α showed distinct patterns, with TNF-α notably elevated in the adult non-T2 group. **(F, G)** Pro-inflammatory and Th17-associated cytokines **(F)** IL-6 and **(G)** IL-17A were analyzed to assess non-type 2 inflammation. **(H–L)** Analysis of serum cytokine levels in the pediatric (n = 142 T2-high, n = 50 non-T2) and adult (n = 103 T2-high, n = 111 non-T2) cohorts. Patients were stratified into T2-high and non-T2 groups based on IgE levels (high IgE, ≥100 IU/mL; low IgE, <100). **(H–K)** Comparison of serum concentrations for **(H)** IL-4, **(I)** IL-9, **(J)** IL-2, and **(K)** IFN-γ. **(L)** Serum cytokine-based odds ratios (ORs) for T2-high versus Non-T2 asthma in pediatric and adult cohorts. Serum concentrations of T2-related and non-T2 cytokines were measured at baseline in a pediatric asthma cohort and an adult asthma cohort, and their associations with T2 inflammatory status were evaluated by logistic regression. The forest plot displays ORs for T2-high versus non-T2 asthma for each analyte (IgE, IL-5, IL-13, IL-2, IL-6, IL-9, IL-10, IFN-γ, TNF-α, IL-17A, IL-17F, IL-4, and IL-22), and the multivariable terms IL-2 (multivariate) and IgE (multivariate), plotted on a linear scale from 0.9 to 1.1. Filled circles indicate estimated ORs, horizontal lines represent 95% confidence intervals, and the vertical dotted line denotes an OR of 1.0 (no association). Across both cohorts, ORs for individual cytokines clustered closely around 1.0, indicating only modest differences in circulating cytokine levels between T2-high and non-T2 patients. Data are presented as dot plots representing individual patient values, with horizontal bars indicating the geometric mean. Statistical significance was determined using the Mann–Whitney *U* test (*p*-values as indicated in the main text). T2-high, type 2-high inflammation; non-T2, non-type 2 inflammation. ns, not significant. * *p* < 0.05, ** *p* < 0.01, and ***p < 0.001.

Next, systemic cytokine profiles were compared between T2-high and non-T2 asthma across the combined pediatric and adult cohorts (**Figures 2A–G**). When all 406 subjects were analyzed together (non-T2, n = 161; T2-high, n = 245), the serum concentrations of the canonical T2 cytokines IL-4 and IL-9 were significantly lower in the non-T2 group than in T2-high patients (**Figures 2A, B**; *p* < 0.05 for both comparisons), whereas TNF-α was also modestly but significantly enriched in T2-high asthma (**Figure 2E**; *p* < 0.05). In contrast, the circulating levels of IL-2, IFN-γ, IL-6, and IL-17A did not differ significantly between T2-high and non-T2 asthma when the cohorts were pooled (**Figures 2C, D, F, G**).

Stratification by age revealed distinct, cohort-specific patterns. In the pediatric cohort, children with non-T2 asthma displayed a prominent non-T2 inflammatory signature, with significantly higher serum IL-2, IFN-γ, IL-6, and IL-17A compared with T2-high children (**Figures 2C, D, F, G**; *p* < 0.05 for all indicated comparisons). Pediatric IL-4 and IL-9 concentrations were numerically higher than in adults but showed no consistent differences between T2 strata. In the adult cohort, the levels of IL-2, IFN-γ, IL-6, and IL-17A were uniformly low and did not differ by T2 status, whereas IL-9 was selectively increased in T2-high adults relative to non-T2 adults (**Figure 2B**; *p* < 0.05). Together, these data indicate that systemic IL-4, IL-9, and TNF-α skewing characterizes T2-high asthma overall, while non-T2 pediatric asthma is marked by elevated Th1/Th17-associated and pro-inflammatory cytokines that are largely absent in adults.

To disentangle the contributions of allergic sensitization from T2-inflammatory status on serum total IgE levels, we next stratified all asthma patients by total serum IgE (high IgE, ≥100 IU/mL; low IgE, <100 IU/mL), generating four groups: non-T2_low IgE (n = 134), T2-high_low IgE (n = 23), non-T2_high IgE (n = 27), and T2-high_high IgE (n = 222) (**Figures 2H–K**). IL-4 and IL-9 concentrations were uniformly low and comparable between the two low-IgE subgroups, whereas both cytokines were substantially higher in patients with high IgE, with the greatest dispersion observed in the T2-high_high IgE group; however, IL-4 and IL-9 levels did not differ significantly between the non-T2_high and T2-high_high IgE subgroups (ns), indicating that elevated IgE rather than T2 status primarily tracked with systemic IL-4 and IL-9 abundance (**Figures 2H, I**). In contrast, IL-2 showed a distinct pattern: within the high-IgE stratum, IL-2 concentrations were significantly higher in non-T2_high IgE patients compared with T2-high_high IgE patients (*p* < 0.05), while both low-IgE subgroups exhibited similarly low IL-2 levels (**Figure 2J**). IFN-γ concentrations tended to be higher in the non-T2_high IgE subjects than in T2-high_high IgE subjects, but this difference did not reach statistical significance (ns) (**Figure 2K**). These findings suggest that high IgE is associated with increased circulating IL-4 and IL-9 irrespective of T2 classification, whereas non-T2_high IgE asthma preferentially retains a Th1/IL-2-skewed cytokine profile.

In univariate analysis, higher IgE levels, whether continuous (OR: 0.986, 95% CI: 0.983–0.989, *p* < 0.0001) or categorical (OR: 0.019, 95% CI: 0.010–0.035, *p* < 0.0001), were inversely associated with non-T2 asthma, while higher IL-2 levels (OR: 1.032, 95% CI: 1.007–1.058, *p* = 0.0119) were positively associated. In multivariate analysis, IgE, whether continuous (OR: 0.992, 95% CI: 0.988–0.995, *p* < 0.0001) or categorical (OR: 0.019, 95% CI: 0.009–0.041, *p* < 0.0001), was independently inversely associated with non-T2 asthma; IL-2 was independently positively associated, regardless of whether IgE was continuous (OR: 1.030, 95% CI: 1.003–1.057, *p* = 0.027) or categorical (OR: 1.029, 95% CI: 1.002–1.056, *p* = 0.035) (**Figure 2L**). These findings demonstrated lower Th2 cytokine levels and an inverse relationship between IgE, alongside a positive association with IL-2 in non-T2 asthma.

### 3.5 WGCNA highlights downregulation of total IgE and IL-4-associated gene module in non-T2 asthma

**Figure 3:**
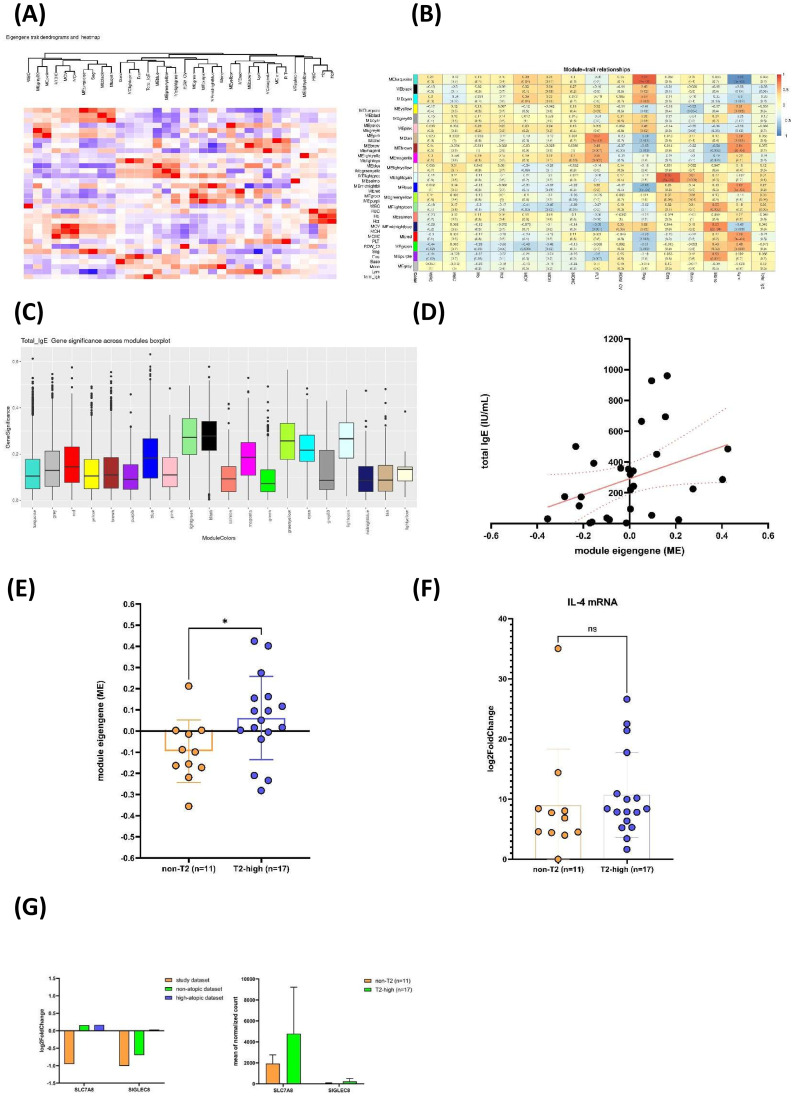
Weighted Gene Co-expression Network Analysis (WGCNA) identifies IgE-associated modules showing lower eigengene expression in non-T2 asthma. **(A)** Eigengene–trait dendrogram heatmap of module–trait relationships. This heatmap represents the hierarchical clustering of module eigengenes and their correlation with clinical traits. Modules, identified by their distinct colors, are hierarchically clustered based on eigengene similarity, with correlation values shown in a gradient from blue (negative correlation) to red (positive correlation). Key relationships highlight significant associations between specific modules and clinical traits. **(B)** Heatmap of module–trait relationships. This matrix displays Pearson’s correlation coefficients and *p*-values (in parentheses) between module eigengenes and clinical traits. **(C)** Gene significance across WGCNA modules for total IgE. The boxplot displays the distribution of gene significance values for total IgE across different WGCNA modules, each represented by a unique module color. Gene significance reflects the correlation between individual genes within each module and total IgE levels. Modules with higher median gene significance values indicate a stronger association with total IgE. Each box represents the interquartile range (IQR), with the horizontal line marking the median, whiskers extending to 1.5× the IQR, and dots representing outliers. The module colors are labeled on the x-axis, and gene significance values are plotted on the y-axis. **(D)** Correlation between lightgreen module eigengene (ME) and total IgE levels. A scatter plot illustrating the linear relationship between the lightgreen ME and serum total IgE levels across the study population. The positive correlation (*r* = 0.83, *p* < 0.001) supports the association of this module with total IgE. **(E)** ME of lightgreen module comparison between non-T2 and T2-high asthma. Box plot comparing the ME between non-T2 (n = 11) and T2-high (n = 17) asthma groups. The significant difference (*p* < 0.05) suggests a lower activity of lightgreen module in non-T2 asthma, consistent with its association with IgE. **(F)** IL-4 mRNA expression levels in non-T2 versus T2-high asthma. Bar plot showing log2-transformed fold changes of IL-4 mRNA expression in non-T2 (n = 11) and T2-high (n = 17) groups. No significant difference was observed (ns). **(G)** Differential expression of *SLC7A8* and *SIGLEC8* across datasets and patient groups. The left panel shows log2 fold change of *SLC7A8* and *SIGLEC8* in the study dataset (orange), non-atopic dataset (green), and high-atopic dataset (blue). The right panel displays the mean normalized counts of *SLC7A8* and *SIGLEC8* between non-T2 (n = 11, orange) and T2-high (n = 17, green) groups. *P < 0.05; ns, not significant.

To explore the relationships between gene co-expression with total IgE levels, WGCNA was employed, and module eigengenes (MEs) were compared between our 11 non-T2 and 17 T2-high patients. Total IgE levels clustered with the ME of the blue module, the ME of the greenyellow module, and the ME of the lightgreen module (**Figure 3A**). Genes within the lightgreen module exhibited a positive correlation with total IgE levels (**Figures 3B–D**, **Supplementary Table 1**). The ME value of the lightgreen module was significantly lower in the non-T2 group than in the T2-high group, suggesting that total IgE was downregulated in non-T2 asthma (**Figure 3E**). Notably, *IL-4*, a key Th2 cytokine, was identified in the lightgreen module, and its mRNA levels were lower in the non-T2 group (**Figure 3F**). WGCNA revealed a total IgE-associated gene module, suggesting downregulated IgE and IL-4 mRNA in non-T2 asthma.

In the overlapped lightgreen module genes with genes from all seven GSEA gene sets, we identified *SLC7A8* and *SIGLEC8*. *SLC7A8* was downregulated in our study dataset and in the non-T2 group. *SIGLEC8* was downregulated in the study dataset and in the non-atopic dataset. It also showed lower expression in the non-T2 group (**Figure 3G**). *SLC7A8* plays a crucial role in type 2 innate lymphoid cells (ILC2) differentiation and activation (28). *SIGLEC8* is highly expressed on eosinophils and mast cells in the airways of patients with asthma, particularly in T2-high disease (29). Our findings implied impaired Th2 differentiation and activation in non-T2 asthma.

### 3.6 Differential activation status of the FcϵRI and BCR reactome pathways in non-T2 dataset

**Figure 4:**
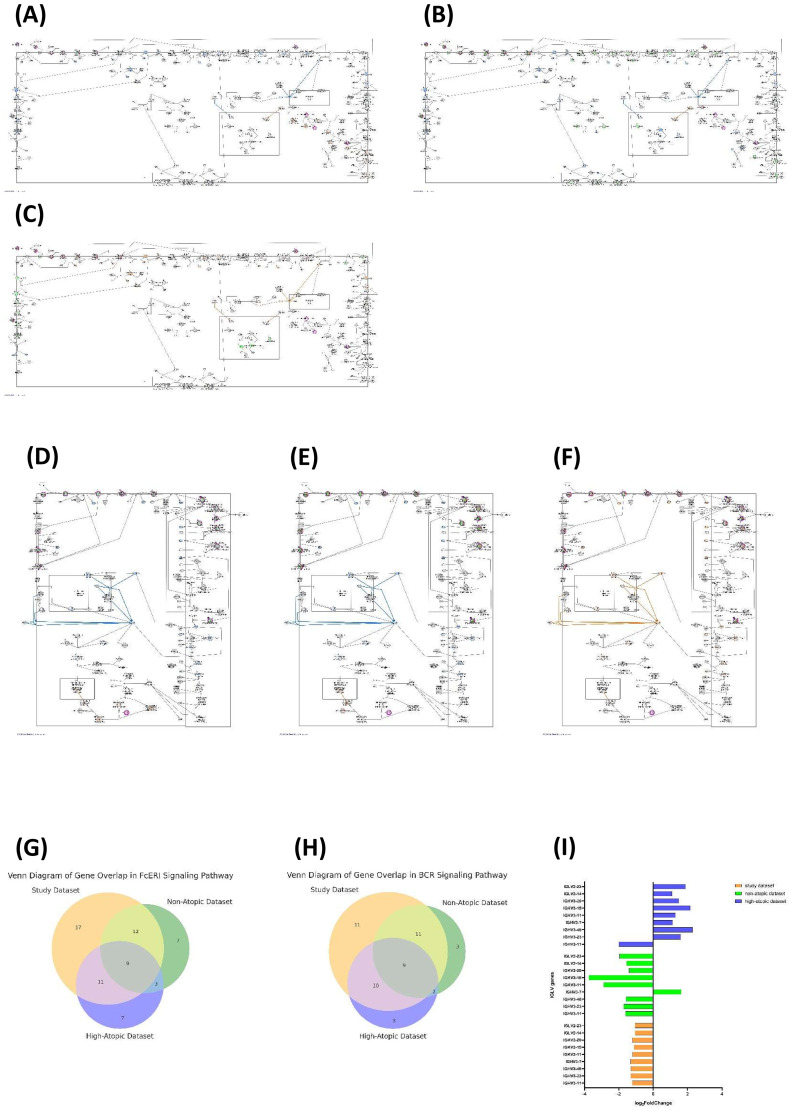
Dataset-specific modulation of high-affinity immunoglobulin E receptor (FcϵRI) and B-cell receptor (BCR) signaling Reactome pathways reveals differential activation status and common gene overlap. This figure illustrates the Reactome pathway analysis results for the high-affinity immunoglobulin E receptor (FcϵRI) signaling pathway across three datasets: study dataset, non-atopic dataset, and high-atopic dataset. Each diagram maps the activation and inhibition of key molecular components and interactions within the pathway derived from each dataset. Nodes are color-coded to represent expression changes, with red denoting more extreme upregulation and green denoting more extreme downregulation, transitioning to lighter shades for less intense regulation. Predicted activation is represented in orange, while predicted inhibition is shown in blue, with gradient intensities reflecting confidence levels in these predictions. Connecting arrows illustrate predicted relationships, with orange arrows indicating activation, blue arrows indicating inhibition, yellow lines representing findings inconsistent with the predicted state of the downstream molecule, and gray lines indicating effects that are not predicted. **(A)** FcϵRI signaling Reactome pathway analysis for the study dataset. **(B)** FcϵRI signaling Reactome pathway analysis for the non-atopic dataset. **(C)** FcϵRI signaling Reactome pathway analysis for the high-atopic dataset. The study dataset demonstrates robust activation of upstream and downstream components in FcϵRI signaling, as indicated by widespread red nodes (upregulated molecules) and orange arrows (predicted activation). Compared to high-atopic dataset, the non-atopic dataset exhibits partial attenuation of the pathway, with green nodes indicating downregulation or inhibition of IgE and IgE receptor components and some blue nodes showing predicted inhibition of downstream molecules. **(D)** BCR signaling pathway in the study dataset. **(E)** BCR signaling pathway in the non-atopic dataset. **(F)** BCR signaling pathway in the high-atopic dataset. In both the study dataset and non-atopic dataset, the BCR signaling pathway shows a reduction in activation of upstream molecules (green nodes), particularly in antigen recognition and early signaling events. The effector responses, such as calcium mobilization, were all predicted to be inhibited in both non-atopic dataset (blue nodes and arrows) but predicted to be activated in high-atopic dataset (orange nodes and arrows). **(G)** Gene overlap in the FcϵRI signaling pathway visualized using a Venn diagram. Datasets include study, non-atopic, and high-atopic. Numbers represent the count of shared genes between datasets. **(H)** Gene overlap in the BCR signaling pathway depicted using a Venn diagram. Datasets include study dataset, non-atopic dataset, and high-atopic dataset. Numbers within each section represent the number of genes shared between the respective datasets. **(I)** Comparison of gene expression (mean normalized counts) from nine overlapped genes between non-T2 (n = 11) and T2-high (n = 17) groups. Error bars indicate standard deviation.

Given their central role in allergic sensitization, we next focused on the pathway activities of signaling by BCR (R-HSA-983705) and FcϵRI signaling (R-HSA-2454202) among the study, non-atopic, and high-atopic datasets (19, 30–32). The pathway activity of the high-atopic dataset was predicted to be activated (FcϵRI signaling: z-score = 5.234, *p* = 5.84 × 10^−6^; signaling by BCR: z-score = 4.098, *p* = 7.97 × 10^−5^), while the pathway activity of the study and non-atopic datasets was predicted to be inhibited (FcϵRI signaling: z-score = −6.856, *p* = 9.83 × 10^−50^; signaling of BCR: z-score = −6.091, *p* = 6.35 × 10^−42^; and FcϵRI signaling: z-score = −3.024, *p* = 2.88 × 10^−3^; signaling of BCR: z-score = −2.60, *p* = 9.24 × 10^−3^, respectively) in FcϵRI (**Figures 4A–C**) and BCR pathways (**Figures 4D–F**). IP3R-mediated Ca^2+^ release from the endoplasmic reticulum and nuclear translocation of the CaN: CaM : NFAT complex were predicted to be upregulated in the high-atopic dataset and downregulated in the study and non-atopic datasets (**Supplementary Figure 4**: FcϵRI-mediated Ca^2+^ mobilization). Although IP3-mediated calcium release, TRPC1-mediated calcium influx, and CRAC channel-mediated calcium influx were predicted to be inhibited in the study and non-atopic datasets, they were predicted to be activated in the high-atopic dataset (**Figures 4D–F**: Cytosolic Ca^2+^ in BCR signaling).

Through IPA canonical pathway analysis, we identified 49, 31, and 30 molecules associated with FcϵRI signaling within the study, non-atopic, and high-atopic datasets, respectively (**Figure 4G**). We also identified 41, 25, and 24 molecules associated with BCR signaling within the study, non-atopic, and high-atopic datasets, respectively (**Figure 4H**). The Venn diagram revealed that nine gene symbols (*IGHV3-11*, *IGHV3-23*, *IGHV3-48*, *IGHV3-7*, *IGKV3-11*, *IGKV3-15*, *IGKV3-20*, *IGLV2-14*, and *IGLV2-23*) were common to all three datasets and identical in both the BCR and FcϵRI signaling. The expression of these nine genes from the high-atopic dataset was upregulated, while that from both the study and non-atopic datasets was downregulated (**Figure 4I**).

### 3.7 Downregulation of CDR-related *IGHV*, *IGKV*, and *IGLV* gene segments in non-T2 asthma

**Figure 5:**
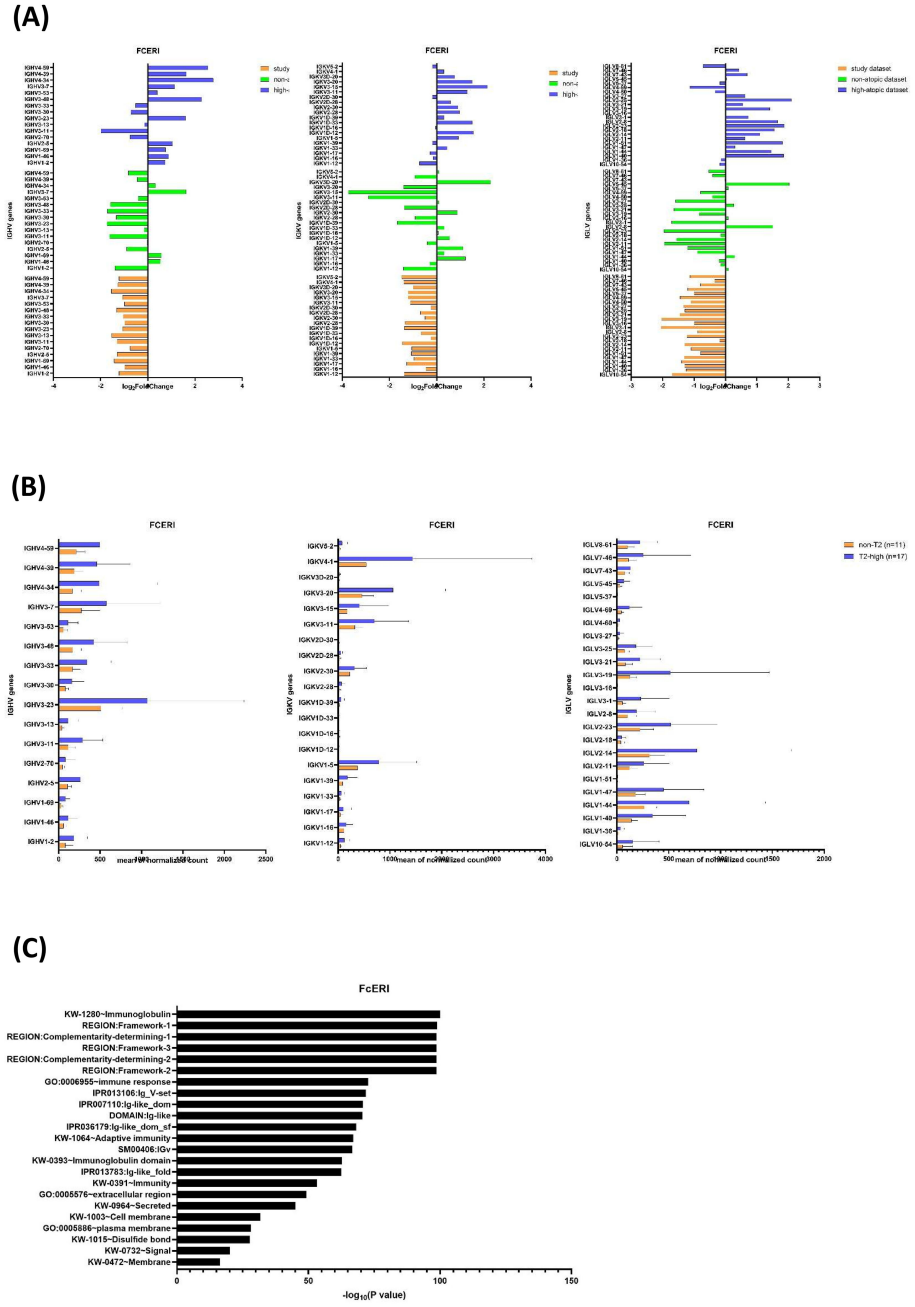
Reduced expression in IgE complementarity-determining regions (CDRs) related to *IGHV*, *IGKV*, and *IGLV* gene segments in non-T2 asthma reveals modulated functional involvement in high-affinity immunoglobulin E receptor (FcϵRI) signaling pathway. **(A)** IgE molecule CDR-related *IGHV*, *IGKV*, and *IGLV* gene expression among three datasets. The high-atopic dataset exhibits the strongest upregulation of FcϵRI-related CDR genes, including *IGHV1–2* and *IGHV3-33*, suggesting a robust activation of IgE-mediated immune pathways. The non-atopic dataset shows a mixed pattern, with both upregulated (e.g., *IGKV3-20*) and downregulated (e.g., *IGKV1-5*) genes, indicating variability in immune signaling responses. The study dataset predominantly displays downregulation of CDR genes, reflecting a more subdued FcϵRI signaling profile compared to the other datasets. **(B)** IgE molecule CDR-related *IGHV*, *IGKV*, and *IGLV* gene expression between non-T2 and T2-high groups. The T2-high group shows significantly higher expression of FcϵRI-related CDR genes, such as *IGKV3–20* and *IGHV3-33*, indicating a stronger IgE-mediated immune response. The non-T2 group has consistently lower expression of these genes, reflecting reduced activation of FcϵRI signaling. **(C)** Database for Annotation, Visualization, and Integrated Discovery (DAVID) for functional annotation clustering of IgE component molecules within the FcϵRI signaling Reactome pathway. The y-axis displays enriched Gene Ontology (GO), KEGG Pathway (KW), InterPro (IPR), SMART domain (SM), and protein region terms. The x-axis represents the statistical significance of enrichment, expressed as −log10(*p*-value). Higher values on the x-axis indicate greater enrichment and statistical significance. The analysis reveals an enrichment for terms related to immunoglobulin structure and function, including *Immunoglobulin*, *Ig_V-set*, *Ig-like_dom*, and *Complementarity-determining region*.

To investigate the molecular mechanisms underlying the lack of allergic sensitization (absence of allergen-specific IgE) in non-T2 asthma, we assessed the expression of immunoglobulin gene segments involved in immune response. BCRs and IgE antibodies share the same pool of V, D, and J gene segments to create their variable regions (and thus can have the same antigen specificity). Thus, we searched the component molecules of the IgE molecule within the FcϵRI signaling Reactome pathway and found 74 molecules (**Supplementary Table 2**). Sixty gene segments corresponded to complementarity-determining regions (CDRs) of immunoglobulin, including 16 immunoglobulin heavy chain variable (*IGHV*) gene segments, 20 immunoglobulin kappa chain variable (*IGKV*) gene segments, and 24 immunoglobulin lambda chain variable (*IGLV*) gene segments, which contributed to the antigen-binding sites (**Supplementary Table 3**). We analyzed the gene expression profiles of component molecules of IgE across three datasets and between patient groups (**Figure 5**). We compared the expression of CDR-related *IGHV*, *IGKV*, and *IGLV* gene segments associated with the IgE molecule across the three datasets (**Figure 5A**). The study dataset showed uniform downregulation (log2 fold change) of CDR-related *IGHV*, *IGKV*, and *IGLV* gene segments. In the non-atopic dataset, most of these gene segments were markedly downregulated. Conversely, the high-atopic dataset showed considerable upregulation of CDR-related *IGHV*, *IGKV*, and *IGLV* gene segments. When comparing our non-T2 and T2-high patient groups, the normalized mean expression of the CDR-related *IGHV*, *IGKV*, and *IGLV* gene segments was higher in the T2-high group than in the non-T2 group (**Figure 5B**). Therefore, the differential abundance of transcripts for variable region-specific gene segments reflects a distinct expression pattern of CDR-related *IGHV*, *IGKV*, and *IGLV* gene segments in non-T2 *vs*. T2-high asthma.

These CDR-related *IGHV*, *IGKV*, and *IGLV* gene segments represent specific functional annotations (**Figure 5C**). Reduced function of *REGION: Framework-1-*, *REGION: Framework-2-*, *REGION: Framework-3-*, *KW-0393-Immunoglobulin domain*, and *GO:0002377~immunoglobulin production* may be associated with decreased IgE stability or expression in non-T2 asthma. Reduced function of *GO:0003823~antigen binding*, *REGION: Complementarity-determining-1-*, *REGION: Complementarity-determining-2-*, and *REGION: Complementarity-determining-3-*, and *GO:0006955~immune response* may imply altered affinity of CDRs for allergens, leading to minimal or no allergen-specific IgE production (negative sensitization) observed in non-T2 asthma. Our findings provided a potential molecular explanation for the absence of allergen-specific IgE, implying limited antigen specificity of IgE in allergic response in non-T2 asthma.

## 4 Discussion

Pathway analysis reveals that Reactome pathways involving the FcϵRI complex and the BCR complex were inhibited. GSEA consistently demonstrated broad suppression of downstream signaling pathways critical for humoral immune activation, including Fcγ receptor-dependent phagocytosis, FcϵRI signaling, and BCR signaling. Dendritic cells (DCs) are known to express a range of receptors associated with scavenger receptors, the complement cascade, Fcγ receptor-mediated phagocytosis, Fcϵ receptor signaling, and innate-adaptive communication (33). During allergen sensitization, tissue-resident DCs capture and process antigens, initiating a cascade of events that culminates in T helper cell polarization (2). We propose that attenuated receptor-mediated signaling in DCs was linked to altered activation states, potentially impacting the differentiation of Th2 cells and follicular helper T (Tfh) cells.

In the non-T2 dataset and patient group, CDR-related *IGHV*, *IGKV*, and *IGLV* genes were downregulated compared to the T2-high dataset and patient group. These CDR-related genes are determinants of the antigen-binding specificity and affinity of IgE. The differential expression of the *IGHV*, *IGKV*, and *IGLV* genes reflects quantitative changes in the transcripts of specific variable gene segments. The frequencies of *IGHV*, *IGKV*, or *IGLV* gene usage in IgE-expressing B cells can provide insight into the immune mechanisms underlying allergic diseases. Kerzel et al. reported that IgE transcripts from preschool children with atopic dermatitis (AD) displayed a preferred dominance other than asthma, suggesting a biased usage of the variable gene segment of the immunoglobulin genes (34). A disproportionate usage of certain *IGHV*, *IGKV*, and *IGLV* genes in IgE-expressing B cells suggests clonality or preferential selection driven by allergen-specific stimulation. *IGLV1–51* participates in antigen recognition, influencing antibody specificity and affinity. *IGLV1-51* may encode light chains that pair with the heavy chains of IgE molecules, forming high-affinity IgE antibodies that target specific allergens, such as peanuts (35). These observed expression changes likely reflect critical immunological processes, including i) increased transcription of IgE-specific *IGHV*, *IGKV*, and *IGLV* genes, suggesting the expansion of particular IgE-producing B cells; ii) preferential gene usage, characterized by biased utilization of specific *IGHV*, *IGKV*, and *IGLV* genes; and iii) downregulation of IgE production.

BCRs on allergen-specific naïve B cells recognize and process antigens. This triggers TCR engagement and CD40–CD40L interaction, essential for B-cell maturation. Together with IL-4/IL-13 signaling, these processes lead to class-switch recombination (CSR) and IgE production (18). The downregulation of *IGHV*, *IGKV*, and *IGLV* genes in non-T2 asthma patients and the significantly lower IL-4 mRNA level in the non-T2 asthma than the T2-high asthma group suggest suppression of the germinal center reactions and signaling cascades driving CSR to IgE. IL-9 promotes mast cell growth and survival, bronchial hyperresponsiveness (BHR), mucus cell metaplasia, and airway wall remodeling (36). Decreased pro-inflammatory cytokines (e.g., IL-6, IL-1β, and TNF-α) are associated with mitigated T2 inflammation (37).Our study compared cytokine expression profiles between patients with non-type 2 (non-T2) and T2-high asthma, stratified by age, to elucidate the immunologic mechanisms underlying these distinct endotypes. Our results demonstrate that non-T2 asthma, particularly among children, is characterized by an active and heterogeneous cytokine environment involving overlapping Th2, Th1, and Th17 pathway activation. Elevated IL-4, IL-9, and TNF-α were observed in non-T2 asthma compared with T2-high asthma, while children exhibited higher levels of IL-2, IL-6, IFN-γ, and IL-17A than adults. These findings indicate that non-T2 asthma is not immunologically quiescent but instead represents a dynamic inflammatory state with mixed immune activation. Non-T2 asthma has traditionally been defined by the absence of eosinophilic inflammation and the low expression of canonical Th2 cytokines, yet recent evidence and our current data challenge this dichotomous framework. Mechanistic studies have established that non-T2 asthma involves prominent activation of Th1 and Th17 responses, with key roles for IL-6, IL-17A, and TNF-α in driving neutrophilic inflammation and airway hyperresponsiveness (38). The increased expression of IL-4, IL-9, and IL-13 in our non-T2 children may appear paradoxical but likely reflects that children with severe asthma have elevated ILC2s even when they are non-T2 (low eosinophils) (39). Notably, non-atopic T2-high variants have been described in children, primarily driven by ILC2 activation, underscoring the cellular diversity within this endotype (40).

Our IgE-stratified analysis indicates that the systemic cytokine milieu exerts a differential influence on whether patients fall into a high- versus low-IgE state. IL-4 and IL-9 concentrations closely tracked with total IgE, being uniformly low in both low-IgE subgroups and substantially higher in patients with high IgE, irrespective of T2 classification. This pattern is consistent with the canonical role of IL-4 (and IL-13) as the key “first signal” driving class-switch recombination to IgE in human B cells (41) and with data showing that IL-9 further amplifies IL-4-dependent IgE and IgG production by B cells and promotes mast cell expansion in IgE-driven allergic disease (42). Thus, in our cohort, high serum IL-4/IL-9 appears to reflect an established type 2 cytokine environment that favors sustained IgE production, whereas low concentrations of these cytokines are associated with maintenance of a low-IgE phenotype, even in individuals categorized as T2-high by clinical or biomarker criteria. In contrast, IL-2 and IFN-γ showed a more complex and partially antagonistic relationship with IgE. IL-2 levels were selectively elevated in the non-T2_high IgE subgroup compared with T2-high_high IgE patients, and IFN-γ displayed a similar, albeit non-significant, trend. Th1-associated cytokines such as IL-2 and IFN-γ are known to counter-regulate Th2 responses and to inhibit IL-4-driven IgE synthesis by human B cells (43, 44). Our data suggest that, even within a high-IgE background, the presence of higher IL-2 (and potentially IFN-γ) identifies a subgroup in which IgE production may be at least partially constrained by ongoing Th1-skewed inflammation, whereas T2-high_high IgE patients exhibit a cytokine profile dominated by IL-4/IL-9 with less Th1 counter-regulation. Collectively, these findings support a model in which the absolute IgE level is not determined by T2 status alone but by the net balance between IgE-promoting Th2/type 2 cytokines (IL-4 and IL-9) and IgE-antagonistic Th1 cytokines (IL-2 and IFN-γ), and they highlight serum cytokine profiling as a potential tool to distinguish qualitatively different high-IgE states in asthma.

A particularly notable observation from our study is the pronounced age-related difference in cytokine patterns. Children with non-T2 asthma exhibited markedly higher levels of IL-6, IFN-γ, and IL-17A than adults, consistent with a Th1/Th17-dominant immune phenotype. This finding aligns with evidence that pediatric airways demonstrate heightened epithelial responsiveness to IL-17A, which contributes to airway inflammation even in well-controlled asthma (45). Our cytokine analysis revealed that pediatric non-T2 asthma is characterized by elevated Th1 and Th17 cytokines, including IL-2, IL-6, IFN-γ, and IL-17A, accompanied by the downregulation of immunoglobulin-associated genes, indicating an attenuated Th2 signature and reduced atopy (20). These cytokine patterns suggest a dominance of innate and Th17-driven inflammation, often triggered by infection or environmental stimuli such as pollution. The elevation of IL-6 and IFN-γ in children suggests that non-T2 asthma in this population is a more inflammatory phenotype, potentially predisposing to frequent exacerbations, accelerated airway remodeling, and corticosteroid resistance. Mechanistically, IL-6-driven STAT3 signaling has been implicated in steroid resistance and persistent airway inflammation in Th17-skewed asthma models (46), reinforcing the clinical importance of these cytokine patterns. Our findings are consistent with multiple recent investigations emphasizing the complexity of non-T2 asthma. Studies have shown that Th17-associated cytokines, including IL-17A and IL-21, are upregulated in neutrophilic and corticosteroid-resistant asthma (47). These insights underscore that non-T2 asthma is driven by multiple overlapping immune pathways rather than a lack of inflammation. Together with our findings, these studies support a view of asthma as a continuous immunologic spectrum rather than discrete Th2-high and Th2-low categories. In adults, non-T2 asthma typically presents as a chronic, neutrophilic, or mixed inflammatory phenotype associated with obesity, smoking, or recurrent infection (11, 48). Elevated IL-6, IL-8, IL-17, and TNF-α suggest sustained activation of Th1/Th17 and inflammasome pathways. Unlike the pediatric form, which reflects immune immaturity and transient environmental influence, adult non-T2 asthma often develops secondary to metabolic dysregulation and prolonged corticosteroid exposure. This phenotype exhibits poor corticosteroid responsiveness and greater comorbidity burden (49). In contrast, adult T2-high asthma remains dominated by Th2 cytokines (IL-4, IL-5, and IL-13) and eosinophilia, showing robust response to biologics and corticosteroids (50). Despite these apparent differences, both the pediatric and adult non-T2 groups exhibited similar clinical severity and exacerbation frequency, suggesting that non-T2 inflammation can be equally pathogenic even without T2 biomarker elevation (51).

Age-stratified analyses revealed that the immunologic correlates of T2 status are fundamentally different in children and adults. In the pediatric cohort, non-T2 asthma was characterized by higher circulating IL-2, IFN-γ, IL-6, and IL-17A compared with T2-high disease, whereas these same Th1/Th17-associated and pro-inflammatory cytokines were uniformly low and non-discriminatory in adults. This pattern is consistent with the emerging view that pediatric non-T2 asthma often reflects a Th1/Th17-skewed, neutrophil-predominant endotype, in contrast to the classic Th2/IL-4/IL-5/IL-13-driven phenotype that dominates many adult cohorts (36). The observation that systemic Th1/Th17 cytokines segregated with non-T2 status only in children suggests that age-related factors—such as developmental immunology, infection burden, obesity, or epigenetic programming—may amplify non-T2 pathways earlier in life and become less prominent or more compartmentalized to the airways with aging (52). In contrast, adult T2-high asthma was distinguished primarily by increased IL-9, with relatively modest differences in other serum cytokines, aligning with reports that a subset of adult T2 asthma is dominated by epithelial–ILC2–B-cell crosstalk and mast cell activation rather than broad systemic Th2 polarization (53). Together, these findings highlight that the same T2/non-T2 classification captures biologically distinct endotypes across the life course and underscore the need for age-specific biomarker cut-offs and therapeutic strategies.

The differential activities of the FcϵRI and BCR Reactome pathways between the non-T2 and T2-high phenotypes correlated with the differential expression of CDR-related *IGHV*, *IGKV*, and *IGLV* genes. The top-ranked canonical pathway identified in the non-T2 study dataset was consistently observed across all subsequent datasets of GSE145505. This universality suggests that these pathways are fundamental to the core immunological framework spanning diverse atopic phenotypes, from non-atopic (non-T2) states to highly atopic (T2-high) conditions. We speculate that a unified regulatory network made up of these Reactome pathways is a key driver of allergic responses, as evidenced by their consistent involvement and dose-dependent regulation across all datasets.

The clinical implications of these results are substantial. First, they highlight the inadequacy of the current binary T2-high/T2-low framework, suggesting that a more nuanced classification incorporating Th1 and Th17 activity is necessary for accurate endotyping and therapy selection. Second, the lack of targeted therapies for non-T2 asthma remains a major unmet need. Unlike T2-high asthma, which benefits from anti-IgE, anti-IL-5, and anti-IL-4Rα biologics, non-T2 asthma lacks effective biologics and is often refractory to corticosteroids (54). The increased Th17 and IL-6/STAT3 signaling observed in our study suggests that inhibitors targeting these pathways, such as IL-17 blockers, JAK inhibitors, or anti-IL-6 agents, could provide novel therapeutic options. Indeed, ruxolitinib, a JAK inhibitor, has shown efficacy in reducing airway hyperresponsiveness and inflammation in corticosteroid-resistant, Th1/Th17-driven asthma models (55).

## 5 Conclusion

Our findings elucidated the inflammatory endotype associated with low total IgE levels and negative sensitization in non-T2 asthma. Non-T2 asthma is characterized by inhibited profiles of canonical pathways related to receptor-mediated pathways. The downregulation of immunoglobulin gene segments like *IGHV*, *IGKV*, and *IGLV* reflects quantitative changes in the abundance of transcripts for specific variable gene segments. The reduced levels of IL-4 and IL-9 in the non-T2 asthma imply diminished Th2 and Tfh cell support for IgE class-switch and allergic response.

## 6 Limitation

There were several limitations that should be acknowledged. First, although future studies will aim to expand the primary sample size, the alignments with empirical benchmarks for bulk RNA-seq indicate that six to 12 biological replicates are typically sufficient to detect differential expression, and employment of the statistical method DESeq2 optimized for smaller datasets, alongside systems-level pathway analyses, supports the reproducibility and generalizability of the reported results. Second, the cytokine analysis relied on their spontaneous serum levels, which may not fully reflect local airway inflammation dynamics. Third, using public GEO datasets for validation is a best practice in systems transcriptomics because it enhances reproducibility, generalizability, and transparency. However, researchers must carefully control for heterogeneity, batch effects, and metadata inconsistencies to ensure reliable validation. Fourth, while we identified several inhibited canonical pathways and differentially expressed genes, IPA provides *inferences*, not direct measurements. The functional validation of these findings in experimental models was not performed, limiting causal interpretation of the results.

## Data Availability

The original contributions presented in the study are included in the article/**Supplementary Material**. Further inquiries can be directed to the corresponding author.

## Glossary

BCR: B-cell receptor
CDR: complementarity-determining region
CSR: class-switch recombination
DAVID: Database for Annotation Visualization, and Integrated Discovery
DCs: dendritic cells
FcϵRI: high-affinity immunoglobulin E receptor
GSEA: Gene Set Enrichment Analysis
ICS: inhaled corticosteroid
IL-10: interleukin-10
IL-13: interleukin-13
IL-17: interleukin-17
IL-17A: interleukin-17A
IL-17F: interleukin-17F
IL-2: interleukin-2
IL-22: interleukin-22
IL-4: interleukin-4
IL-5: interleukin-5
IL-6: interleukin-6
IL-9: interleukin-9
ILC2s: type 2 innate lymphoid cells
IPA: Ingenuity Pathway Analysis
LAT2: linker for activation of T-cell family member 2
LYN: lymphocyte-specific protein tyrosine kinase
MAPK: mitogen-activated protein kinase
MCs: mast cells
ME: module eigengene
MHC: major histocompatibility complex
NF-κB: nuclear factor kappa B
NTAL: non-T cell activation linker
OR: odds ratio
PLC: phospholipase C
RAST: radioallergosorbent test
RNA-seq: RNA sequencing
SAS: Statistical Analysis Software
SPT: skin prick test
Syk: spleen tyrosine kinase
T2: type 2
T2-high: type 2 high inflammation
T2-low: type 2 low inflammation
TCR: T-cell receptor
Th9: T helper cell type 9
Tfh: follicular helper T cell
TOM: topological overlap matrix
TRIM21: tripartite motif containing 21
TRPC1: transient receptor potential cation channel subfamily C member 1
WGCNA: Weighted Gene Co-Expression Network Analysis

## References

[1] Fahy JV . Type 2 inflammation in asthma--present in most, absent in many. Nat Rev Immunol. (2015) 15:57–65. doi: 10.1038/nri3786, PMID: 25534623 PMC4390063

[2] Hammad H Lambrecht BN . The basic immunology of asthma. Cell. (2021) 184:1469–85. doi: 10.1016/j.cell.2021.02.016, PMID: 33711259

[3] Reddel HK Bateman ED Schatz M Krishnan JA Cloutier MM . A practical guide to implementing SMART in asthma management. J Allergy Clin Immunology: In Practice. (2022) 10:S31–S8. doi: 10.1016/j.jaip.2021.10.011, PMID: 34666208

[4] Rackemann FM . A working classification of asthma. Am J Med. (1947) 3:601–6. doi: 10.1016/0002-9343(47)90204-0, PMID: 20269240

[5] Romanet-Manent S Charpin D Magnan A Lanteaume A Vervloet D . Allergic vs nonallergic asthma: what makes the difference? Allergy. (2002) 57:607–13. doi: 10.1034/j.1398-9995.2002.23504.x, PMID: 12100301

[6] Novak N Bieber T . Allergic and nonallergic forms of atopic diseases. J Allergy Clin Immunol. (2003) 112:252–62. doi: 10.1067/mai.2003.1595, PMID: 12897728

[7] McIntyre AP Viswanathan RK . Phenotypes and endotypes in asthma. In: Precision Approaches to Heterogeneity in Asthma. Cham, Switzerland: Springer (2023). p. 119–42., PMID: 10.1007/978-3-031-32259-4_6 37464119

[8] Foppiano F Schaub B . Childhood asthma phenotypes and endotypes: a glance into the mosaic. Mol Cell Pediatr. (2023) 10:9. doi: 10.1186/s40348-023-00159-1, PMID: 37646843 PMC10469115

[9] Kyriakopoulos C Gogali A Bartziokas K Kostikas K . Identification and treatment of T2-low asthma in the era of biologics. ERJ Open Res. (2021) 7 (2):00309–2020. doi: 10.1183/23120541.00309-2020, PMID: 34109244 PMC8181790

[10] Fitzpatrick AM Chipps BE Holguin F Woodruff PG . T2-”Low” Asthma: overview and management strategies. J Allergy Clin Immunol In practice. (2020) 8:452–63. doi: 10.1016/j.jaip.2019.11.006, PMID: 32037109

[11] Ricciardolo FLM Sprio AE Baroso A Gallo F Riccardi E Bertolini F . Characterization of T2-low and T2-high asthma phenotypes in real-life. Biomedicines. (2021) 9 (11):1684. doi: 10.3390/biomedicines9111684, PMID: 34829913 PMC8615363

[12] Korevaar DA Westerhof GA Wang J Cohen JF Spijker R Sterk PJ . Diagnostic accuracy of minimally invasive markers for detection of airway eosinophilia in asthma: a systematic review and meta-analysis. Lancet Respir Med. (2015) 3:290–300. doi: 10.1016/S2213-2600(15)00050-8, PMID: 25801413

[13] Zervas E Samitas K Papaioannou AI Bakakos P Loukides S Gaga M . An algorithmic approach for the treatment of severe uncontrolled asthma. ERJ Open Res. (2018) 4 (1):00125–2017. doi: 10.1183/23120541.00125-2017, PMID: 29531957 PMC5838355

[14] Virchow JC Jr . Intrinsic asthma. Asthma Rhinitis. (2000), 1355–78.

[15] Peters SP . Asthma phenotypes: nonallergic (intrinsic) asthma. J Allergy Clin Immunol In practice. (2014) 2:650–2. doi: 10.1016/j.jaip.2014.09.006, PMID: 25439352

[16] Niessen NM Fricker M McDonald VM Gibson PG . T2-low: what do we know?: Past, present, and future of biologic therapies in noneosinophilic asthma. Ann Allergy Asthma Immunol. (2022) 129:150–9. doi: 10.1016/j.anai.2022.04.020, PMID: 35487388

[17] Akdis CA Arkwright PD Brüggen MC Busse W Gadina M Guttman-Yassky E . Type 2 immunity in the skin and lungs. Allergy. (2020) 75:1582–605. doi: 10.1111/all.14318, PMID: 32319104

[18] Komlósi ZI van de Veen W Kovács N Szűcs G Sokolowska M O’Mahony L . Cellular and molecular mechanisms of allergic asthma. Mol Aspects Med. (2022) 85:100995. doi: 10.1016/j.mam.2021.100995, PMID: 34364680

[19] Nagata Y Suzuki R . FcϵRI: A master regulator of mast cell functions. Cells. (2022) 11:622. doi: 10.3390/cells11040622, PMID: 35203273 PMC8870323

[20] Lee JH Yang YH Lin YT Wang LC Yu HH Hu YC . Characterizing non-T2 asthma: key pathways and molecular implications indicative of attenuated th2 response. Inflammation. (2024) 48 (4):1839–1862. doi: 10.1007/s10753-024-02159-3, PMID: 39466498

[21] Expert panel report 3 (EPR-3): guidelines for the diagnosis and management of asthma-summary report 2007. J Allergy Clin Immunol. (2007) 120:S94–138. doi: 10.1016/j.jaci.2007.09.029, PMID: 17983880

[22] Hogan AD Bernstein JA . GINA updated 2019: Landmark changes recommended for asthma management. Ann allergy Asthma Immunol. (2020) 124:311–3. doi: 10.1016/j.anai.2019.11.005, PMID: 31734328

[23] Altman MC Calatroni A Ramratnam S Jackson DJ Presnell S Rosasco MG . Endotype of allergic asthma with airway obstruction in urban children. J Allergy Clin Immunol. (2021) 148:1198–209. doi: 10.1016/j.jaci.2021.02.040, PMID: 33713771 PMC8429519

[24] Subramanian A Tamayo P Mootha VK Mukherjee S Ebert BL Gillette MA . Gene set enrichment analysis: a knowledge-based approach for interpreting genome-wide expression profiles. Proc Natl Acad Sci U S A. (2005) 102:15545–50. doi: 10.1073/pnas.0506580102, PMID: 16199517 PMC1239896

[25] Mootha VK Lindgren CM Eriksson K-F Subramanian A Sihag S Lehar J . PGC-1α-responsive genes involved in oxidative phosphorylation are coordinately downregulated in human diabetes. Nat Genet. (2003) 34:267–73. doi: 10.1038/ng1180, PMID: 12808457

[26] Yu C Kobeissy F . Systems biology applications to decipher mechanisms and novel biomarkers in CNS trauma. In: Brain Neurotrauma: Molecular, Neuropsychological, and Rehabilitation Aspects. Boca Raton (FL): CRC Press/Taylor & Francis. Frontiers in Neuroengineering. (2015)., PMID: 26269919

[27] Milacic M Beavers D Conley P Gong C Gillespie M Griss J . The reactome pathway knowledgebase 2024. Nucleic Acids Res. (2024) 52:D672–d8. doi: 10.1093/nar/gkad1025, PMID: 37941124 PMC10767911

[28] Panda SK Kim DH Desai P Rodrigues PF Sudan R Gilfillan S . SLC7A8 is a key amino acids supplier for the metabolic programs that sustain homeostasis and activation of type 2 innate lymphoid cells. Proc Natl Acad Sci U S A. (2022) 119:e2215528119. doi: 10.1073/pnas.2215528119, PMID: 36343258 PMC9674248

[29] Kerr SC Gonzalez JR Schanin J Peters MC Lambrecht BN Brock EC . An anti-siglec-8 antibody depletes sputum eosinophils from asthmatic subjects and inhibits lung mast cells. Clin Exp Allergy. (2020) 50:904–14. doi: 10.1111/cea.13681, PMID: 32542913 PMC7610812

[30] Turner H Kinet J-P . Signalling through the high-affinity IgE receptor FcϵRI. Nature. (1999) 402:24–30. doi: 10.1038/35037021, PMID: 10586892

[31] Wang J Zhou Y Zhang H Hu L Liu J Wang L . Pathogenesis of allergic diseases and implications for therapeutic interventions. Signal Transduction Targeted Ther. (2023) 8:138. doi: 10.1038/s41392-023-01344-4, PMID: 36964157 PMC10039055

[32] Liu W Tolar P Song W Kim TJ . Editorial: BCR signaling and B cell activation. Front Immunol. (2020) 11:45. doi: 10.3389/fimmu.2020.00045, PMID: 32063903 PMC6999073

[33] Guermonprez P Valladeau J Zitvogel L Théry C Amigorena S . Antigen presentation and T cell stimulation by dendritic cells. Annu Rev Immunol. (2002) 20:621–67. doi: 10.1146/annurev.immunol.20.100301.064828, PMID: 11861614

[34] Kerzel S Rogosch T Struecker B Maier RF Kabesch M Zemlin M . Unlike in children with allergic asthma, igE transcripts from preschool children with atopic dermatitis display signs of superantigen-driven activation. J Immunol. (2016) 196:4885–92. doi: 10.4049/jimmunol.1402889, PMID: 27183570

[35] Hoh RA Joshi SA Lee J-Y Martin BA Varma S Kwok S . Origins and clonal convergence of gastrointestinal IgE+ B cells in human peanut allergy. Sci Immunol. (2020) 5:eaay4209. doi: 10.1126/sciimmunol.aay4209, PMID: 32139586 PMC7691169

[36] Lambrecht BN Hammad H Fahy JV . The cytokines of asthma. Immunity. (2019) 50:975–91. doi: 10.1016/j.immuni.2019.03.018, PMID: 30995510

[37] Liu T Woodruff PG Zhou X . Advances in non-type 2 severe asthma: from molecular insights to novel treatment strategies. Eur Respir J. (2024) 64 (2):2300826. doi: 10.1183/13993003.00826-2023, PMID: 38697650 PMC11325267

[38] Hudey SN Ledford DK Cardet JC . Mechanisms of non-type 2 asthma. Curr Opin Immunol. (2020) 66:123–8. doi: 10.1016/j.coi.2020.10.002, PMID: 33160187 PMC7852882

[39] Nagakumar P Denney L Fleming L Bush A Lloyd CM Saglani S . Type 2 innate lymphoid cells in induced sputum from children with severe asthma. J Allergy Clin Immunol. (2016) 137:624–6.e6. doi: 10.1016/j.jaci.2015.06.038, PMID: 26277593

[40] Bonato M Bazzan E Snijders D Turato G Turrin M Cosio MG . Innate lymphocytes -ILC2- might be the drivers of T2-high nonatopic asthma in children. Eur Respir J. (2021) 58 (suppl 65):PA861. doi: 10.1183/13993003.congress-2021.PA861

[41] Bacharier LB Geha RS . Molecular mechanisms of IgE regulation. J Allergy Clin Immunol. (2000) 105:S547–S58. doi: 10.1016/S0091-6749(00)90059-9, PMID: 10669540

[42] Yazdani R Shapoori S Rezaeepoor M Sanaei R Ganjalikhani-Hakemi M Azizi G . Features and roles of T helper 9 cells and interleukin 9 in immunological diseases. Allergologia immunopathologia. (2019) 47:90–104. doi: 10.1016/j.aller.2018.02.003, PMID: 29703631

[43] Kurasawa K Iwamoto I . Mechanism and regulation of IgE production in allergy. Nihon Rinsho. (1996) 54:434–9. 8838093

[44] Pene J Rousset F Brière F Chrétien I Bonnefoy J-Y Spits H . IgE production by normal human lymphocytes is induced by interleukin 4 and suppressed by interferons gamma and alpha and prostaglandin E2. Proc Natl Acad Sci. (1988) 85:6880–4. doi: 10.1073/pnas.85.18.6880, PMID: 2970644 PMC282082

[45] McAlees JW Baker T Houshel L McKnight C Lindsley A Strait RT . Increased nasal epithelial cell responsiveness to IL-17A in paediatric asthmatics with low blood neutrophil count, low traffic-related air pollution exposure and good asthma control. Clin Exp Allergy. (2022) 52:569–73. doi: 10.1111/cea.14080, PMID: 34908201 PMC8976742

[46] Xue Y Zhou Y Bao W Fu Q Hao H Han L . STAT3 and IL-6 contribute to corticosteroid resistance in an OVA and ozone-induced asthma model with neutrophil infiltration. Front Pharmacol. (2021) 8:717962. doi: 10.3389/fmolb.2021.717962, PMID: 34760922 PMC8573338

[47] Duan W Huang J Wasti B Chen Z Yuan Y He Y . miR-146a-3p as a potential novel therapeutic by targeting MBD2 to mediate Th17 differentiation in Th17 predominant neutrophilic severe asthma. Clin Exp Med. (2023) 23:2839–54. doi: 10.1007/s10238-023-01033-0, PMID: 36961677 PMC10543568

[48] To M Arimoto Y Honda N Kurosawa Y Haruki K To Y . Clinical characteristics and cytokine profiles of adult obese asthma with type2 inflammation. Sci Rep. (2023) 13 (1):14799. doi: 10.1038/s41598-023-41889-6, PMID: 37684314 PMC10491644

[49] Hudler A Holguin F Sharma S . Type 2 or non–type 2 asthma exacerbations? That is the question. Am Thorac Society;. (2022) p:521–2. doi: 10.1164/rccm.202205-0857ED, PMID: 35584351 PMC9716916

[50] Chheang C Guinand S von Garnier C Sartori C . New perspectives of biological therapy for severe asthma in adults and adolescents. Swiss Med Weekly. (2022) 152:w30176–w. doi: 10.4414/SMW.2022.w30176, PMID: 35748315

[51] McDowell PJ Busby J Hanratty CE Djukanovic R Woodcock A Walker S . Exacerbation profile and risk factors in a type-2–low enriched severe asthma cohort: a clinical trial to assess asthma exacerbation phenotypes. Am J Respir Crit Care Med. (2022) 206:545–53. doi: 10.1164/rccm.202201-0129OC, PMID: 35549845 PMC9716911

[52] Weckmann M Thiele D Liboschik L Bahmer T Pech M Dittrich AM . Cytokine levels in children and adults with wheezing and asthma show specific patterns of variability over time. Clin Exp Immunol. (2021) 204:152–64. doi: 10.1111/cei.13550, PMID: 33202033 PMC7944353

[53] Zhou Y McLane M Levitt RC . Th2 cytokines and asthma — Interleukin-9 as a therapeutic target for asthma. Respir Res. (2001) 2:80. doi: 10.1186/rr42, PMID: 11686869 PMC59572

[54] Peri F Amaddeo A Badina L Maschio M Barbi E Ghirardo S . T2-low asthma: A discussed but still orphan disease. Biomedicines. (2023) 11 (4):1226. doi: 10.3390/biomedicines11041226, PMID: 37189844 PMC10136127

[55] Subramanian H Hashem T Bahal D Kammala A Thaxton K Das R . Ruxolitinib ameliorates airway hyperresponsiveness and lung inflammation in a corticosteroid-resistant murine model of severe asthma. Front Immunol. (2021) 12. doi: 10.3389/fimmu.2021.786238, PMID: 34777398 PMC8586657

