# Supplementary material for: Low IgE and absence of sensitization in non-T2 asthma: a transcriptomic and cytokine study
Source: Front Immunol. 2026 Jan 12;16:1711616. doi: 10.3389/fimmu.2025.1711616 (PMC12832990; doi:10.3389/fimmu.2025.1711616)
Supplement: Supplementary file 1 [file Supplementaryfile1.pdf]

Low IgE and Absence of Sensitization in Non-T2 Asthma: A Transcriptomic and Cytokine Study

Jyh-Hong Lee [1*], Yu-Tsan Lin [1], Li-Chieh Wang [1], Hsin-Hui Yu [1], Ya-Chiao Hu [1], Yao-Hsu Yang [1], Bor-Luen Chiang [1, 2]

[1] Department of Pediatrics, National Taiwan University Hospital and National Taiwan University College of Medicine, Taipei, Taiwan, Republic of China

[2] Graduate Institute of Clinical Medicine, National Taiwan University College of Medicine, Taipei, Taiwan, Republic of China

Correspondence: Jyh-Hong Lee*

Department of Pediatrics, National Taiwan University Hospital, 8 Chung-Shan South Road, Taipei 10002, Taiwan, Republic of China

Tel: 886-2-23123456 ext. 71719; Fax: 886-2-23119087; Email: leonid@ntu.edu.tw

ORCID: 0000-0003-4285-4912

BRIEF INDEX

Supplement Results

Supplemental Figures

Supplement Figure 1. *Comparison of serum levels of IL-5, IL-10, IL-13, IL-17F, and IL-22 between non-T2 and T2-high asthma patients.*

Supplement Figure 2. *IgE-Allergen FcεRI pathway in study, non-atopic, and high-atopic datasets.*

Supplement Figure 3. *FcεRI-mediated MAPK activation in study, non-atopic, and high-atopic datasets.*

Supplement Figure 4. *FcεRI-mediated Ca^{2+} mobilization in study, non-atopic, and high-atopic datasets.*

Supplement Figure 5. *Role of LAT2/NTAL/LAB in calcium mobilization in study, non-atopic, and high-atopic datasets.*

Supplemental Tables

Supplement Table 1. *Genes in lightgreen module with module membership (MM) and gene significance (GS).*

Supplement Table 2. *Component molecules of IgE.*

Supplement Table 3. *Gene symbols associated with CDRs of IgE molecule, categorized into IGHV, IGKV, and IGLV segments.*

SUPPLEMENT RESULTS

3.6 Differential activation status of the FcεRI and BCR reactome pathways in Non-T2 datasets

FcεRI reactome signaling

IgE-Allergen FcεRI pathway

Analysis of the IgE-allergen-FcεRI pathway in the study dataset revealed significant downregulation of multiple steps. These included reduced IgE binding to FcεRI, decreased allergen-induced FcεRI aggregation, and impaired phosphorylation of key signaling molecules such as LYN and SYK (***supplement Figure 2***). Interestingly, the high-atopic dataset showed greater upregulation of IgE expression than the non-atopic dataset, in which there was a balance between upregulation and downregulation.

FcεRI-mediated MAPK activation

We investigated the regulation of FcεRI-mediated MAPK activation in the study, non-atopic, and high-atopic datasets. The study and non-atopic datasets

exhibited a predominant inhibitory profile, with most molecules and complexes involved in this pathway predicted to be downregulated. By contrast, the high-atopic dataset showed a clear activation pattern (***supplement Figure 3***).

FcεRI-mediated Ca^{2+} *mobilization* (***supplement Figure 4***)

FcεRI-mediated NF-κB activation

The study dataset exhibited an activation profile for the NF-κB pathway, suggesting potential upregulation of NF-κB signaling. In contrast, the high-atopic dataset showed downregulation or inhibition of key components involved in NF-κB activation (**Figure 4**).

Function of LAT2/NTAL/LAB in calcium mobilization

The study and non-atopic datasets exhibited a predominant inhibitory profile for this pathway, with most molecules and complexes predicted to be downregulated. The high-atopic dataset showed a clear downregulation pattern, suggesting impaired calcium signaling (***supplement Figure 5***).

SUPPLEMENT FIGURES

supplement Figure 1. Comparison of serum levels of IL-5 (a), IL-10 (b), IL-13 (c), IL-17F (d), and IL-22 (e) between non-T2 (n=161) and T2-high asthma (n=245) patients. We also compared pediatric (*n* = 192) vs adult (*n* = 214) asthma groups. Serum cytokines levels (pg/mL) were quantified using a bead-based multiplex Human Th Cytokine kit. Data are presented as mean ± standard deviation (SD). n.s.: no statistical significance.

(a)

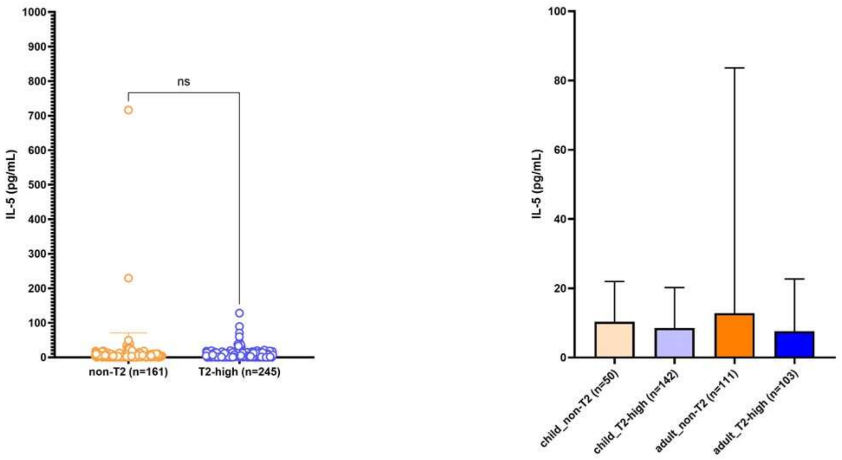

(b)

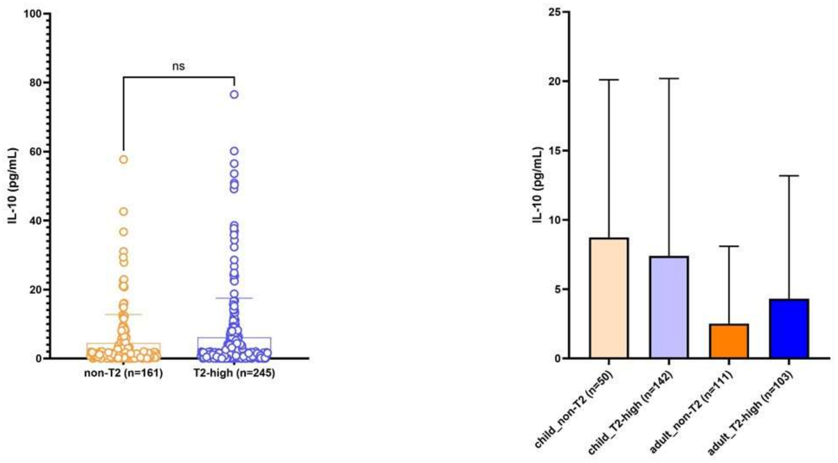
ns
IL-10 (pg/mL)
100
80
60
40
20
0
non-T2 (n=161)
T2-high (n=245)
IL-10 (pg/mL)
25
20
15
10
5
0
child_non-T2 (n=50)
child_T2-high (n=142)
adult_non-T2 (n=111)
adult_T2-high (n=103)

(c)

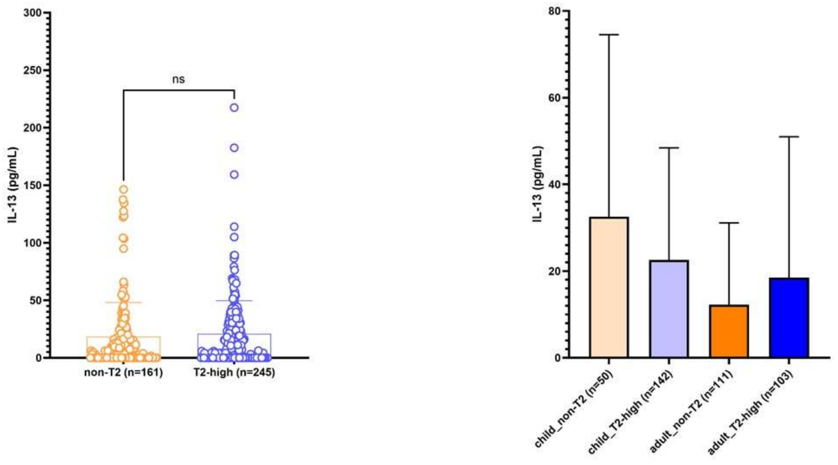
ns
IL-13 (pg/mL)
300
250
200
150
100
50
0
non-T2 (n=161)
T2-high (n=245)
IL-13 (pg/mL)
80
60
40
20
0
child_non-T2 (n=50)
child_T2-high (n=142)
adult_non-T2 (n=111)
adult_T2-high (n=103)

(d)

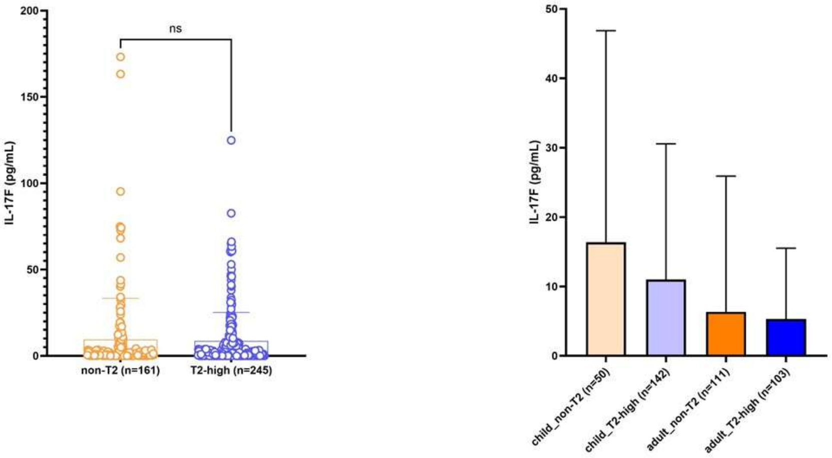
ns
IL-17F (pg/mL)
non-T2 (n=161)
T2-high (n=245)
IL-17F (pg/mL)
child_non-T2 (n=50)
child_T2-high (n=142)
adult_non-T2 (n=111)
adult_T2-high (n=103)

(e)

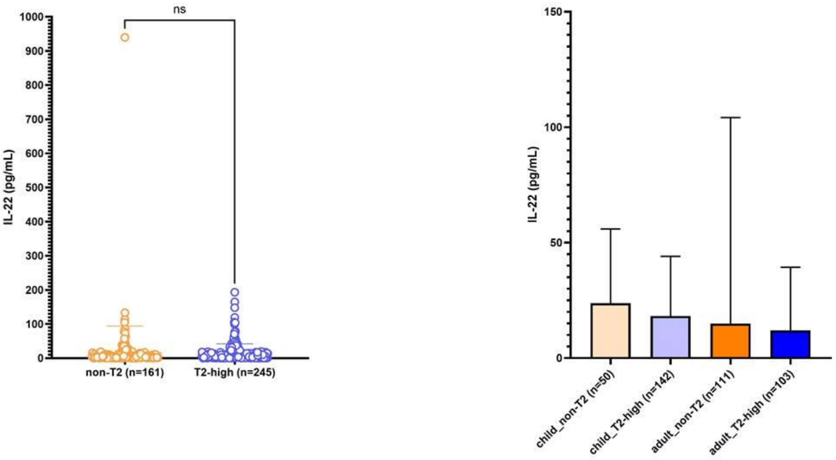
ns
IL-22 (pg/mL)
non-T2 (n=161)
T2-high (n=245)
IL-22 (pg/mL)
child_non-T2 (n=50)
child_T2-high (n=142)
adult_non-T2 (n=111)
adult_T2-high (n=103)

supplement Figure 2. IgE- Allergen FcεRI pathway of study dataset (a), non-atopic dataset (b), and high-atopic dataset (c).

(a)

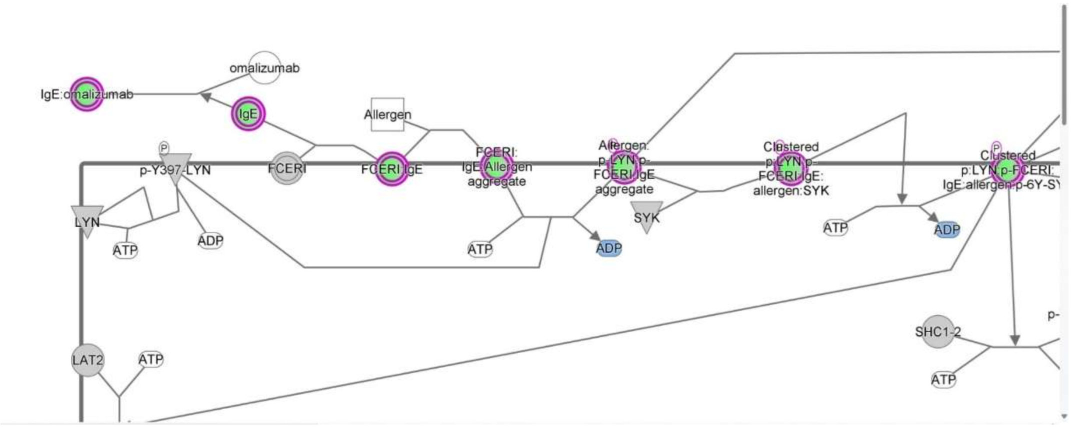

(b)

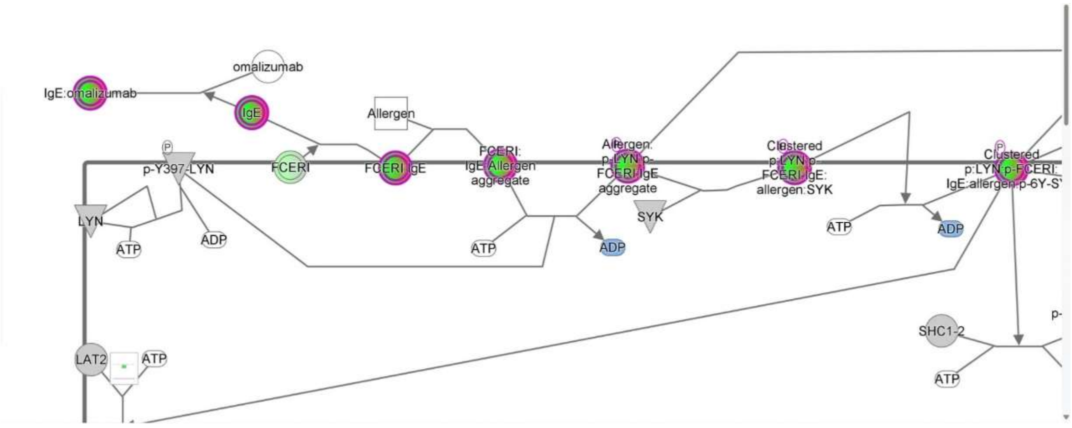

(c)

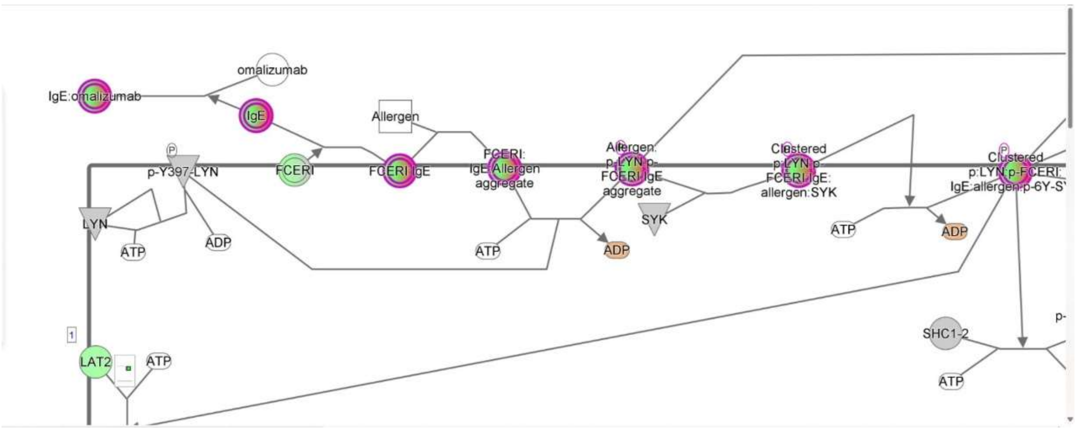

supplement Figure 3. FcεRI mediated MAPK activation (a) of study dataset (b), non-atopic dataset (c), and high-atopic dataset (d).

(a)

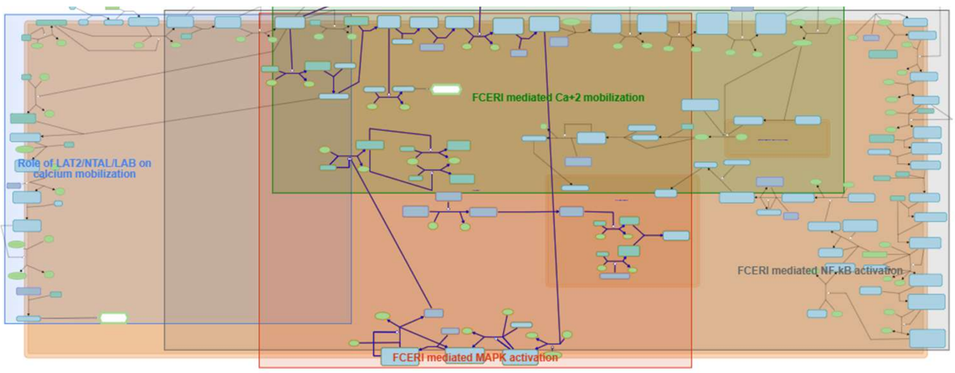

(b)

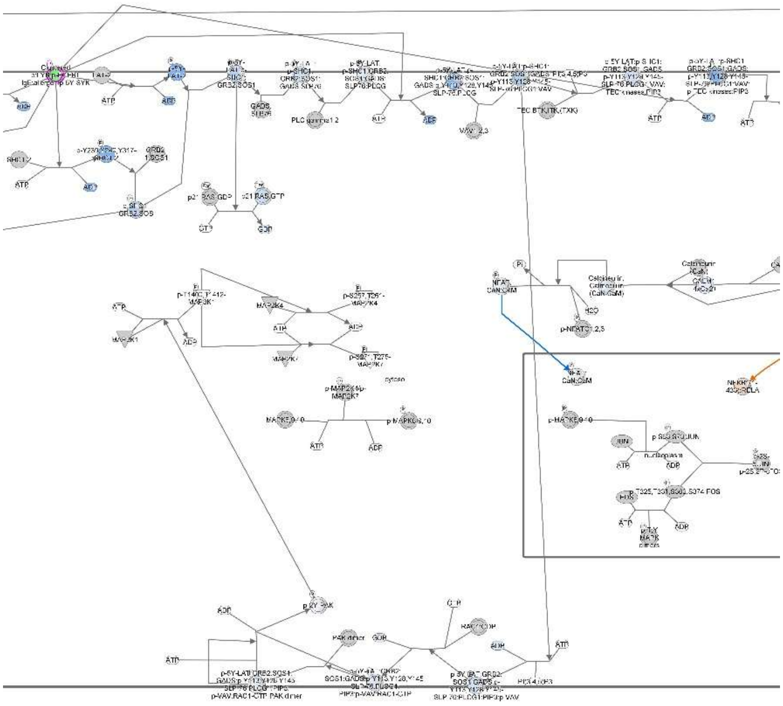

(c)

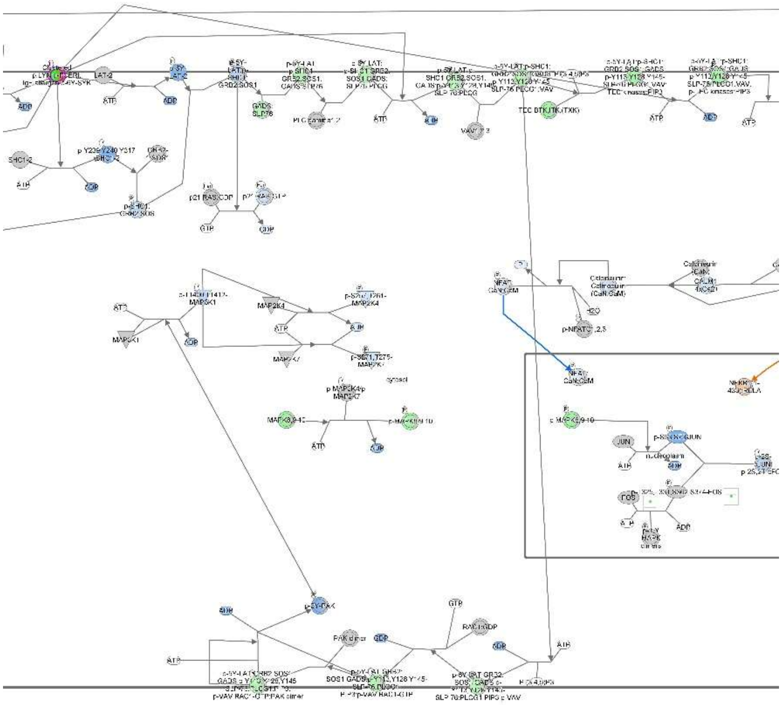

(d)

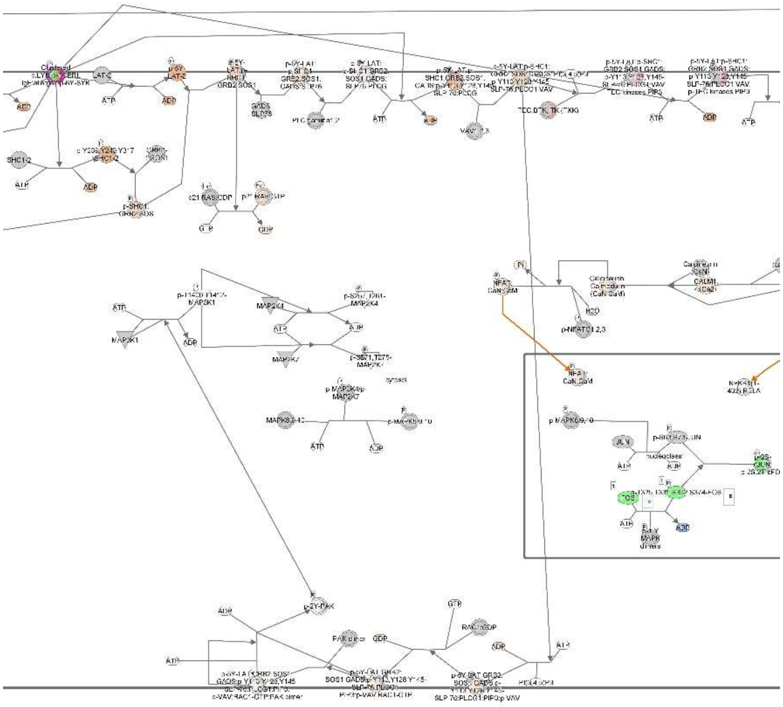

supplement Figure 4. FCERI mediated Ca+2 mobilization (a) of study dataset (b),

non-atopic dataset (c), and high-atopic dataset (d).

(a)

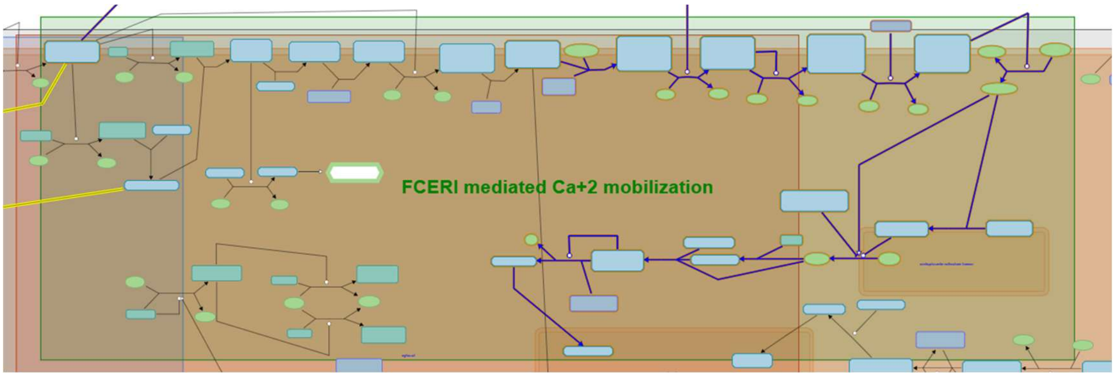

(b)

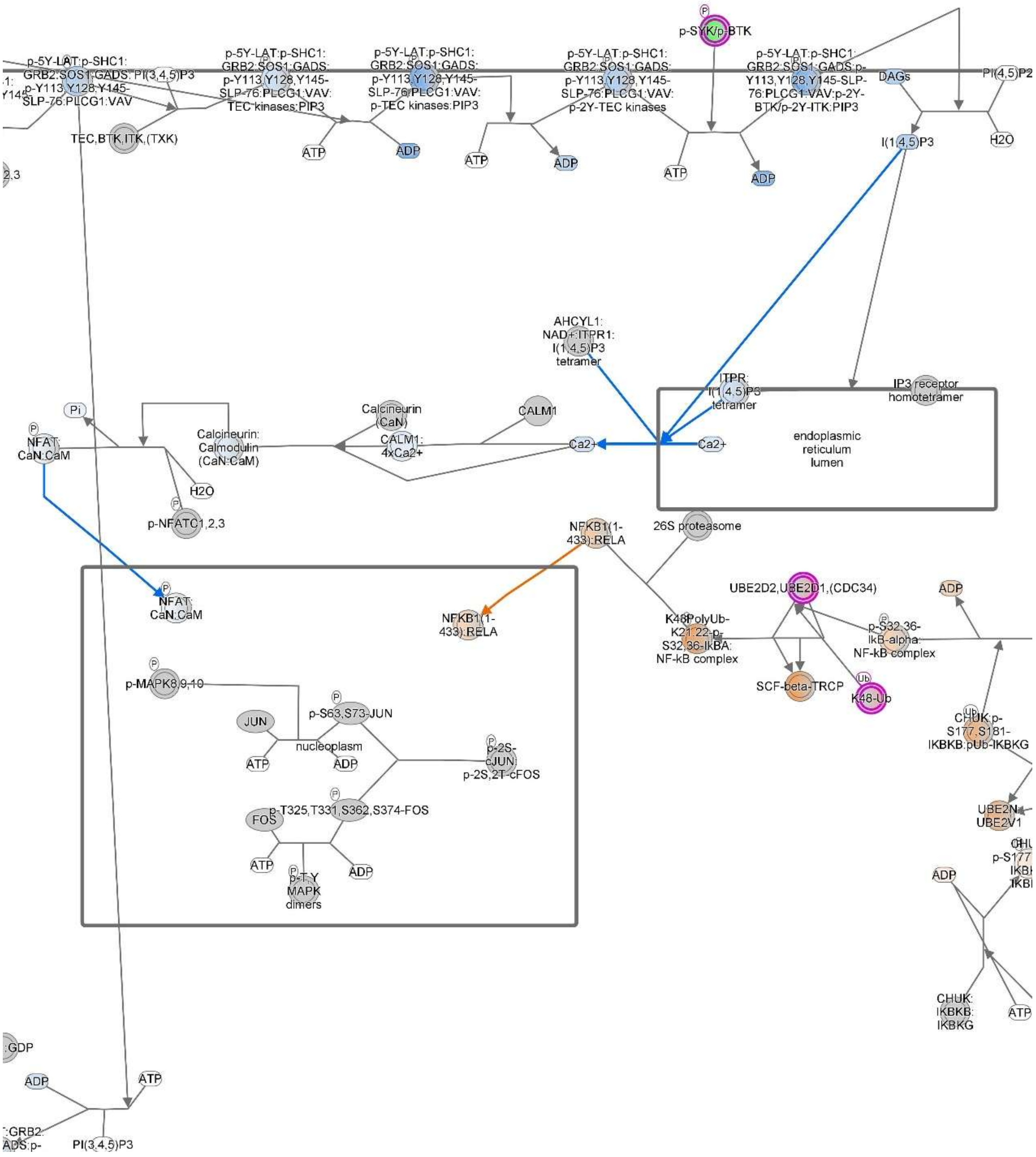
p-SYK/p-BTK
p-5Y-LAT:p-SHC1:
GRB2:SOS1:GADS:PI(3,4,5)P3
p-Y113,Y128,Y145-
SLP-76:PLCG1:VAV
TEC,BTK,ITK,(TXK)
p-5Y-LAT:p-SHC1:
GRB2:SOS1:GADS:
p-Y113,Y128,Y145-
SLP-76:PLCG1:VAV:
TEC kinases:PIP3
p-5Y-LAT:p-SHC1:
GRB2:SOS1:GADS:
p-Y113,Y128,Y145-
SLP-76:PLCG1:VAV:
p-TEC kinases:PIP3
p-5Y-LAT:p-SHC1:
GRB2:SOS1:GADS:
p-Y113,Y128,Y145-
SLP-76:PLCG1:VAV:
p-2Y-TEC kinases
p-5Y-LAT:p-SHC1:
GRB2:SOS1:GADS:p-
Y113,Y128,Y145-SLP-
76:PLCG1:VAV:p-2Y-
BTK/p-2Y-ITK:PIP3
DAGs
PI(4,5)P2
I(1,4,5)P3
H2O
ATP
ADP
AHCYL1:
NAD+:ITPR1:
I(1,4,5)P3
tetramer
ITPR:
I(1,4,5)P3
tetramer
IP3 receptor
homotetramer
endoplasmic
reticulum
lumen
Ca2+
Pi
NFAT:
CaN:CaM
Calcineurin:
Calmodulin
(CaN:CaM)
Calcineurin
(CaN)
CALM1:
4xCa2+
CALM1
H2O
p-NFATC1,2,3
NFKB1(1-
433):RELA
26S proteasome
UBE2D2,UBE2D1,(CDC34)
K48PolyUb-
K21,22-p-
S32,36-IkBA:
NF-kB complex
p-S32,36-
IkB-alpha:
NF-kB complex
SCF-beta-TRCP
K48-Ub
CHUK:p-
S177,S181-
IKBKB:pUb-IKBKG
UBE2N:
UBE2V1
CHUK:
IKBKB:
IKBKG
p-MAPK8,9,10
JUN
p-S63,S73-JUN
nucleoplasm
p-2S-
cJUN:
p-2S,2T-cFOS
FOS
p-T325,T331,S362,S374-FOS
p-T,Y
MAPK
dimers
PI(3,4,5)P3

(c)

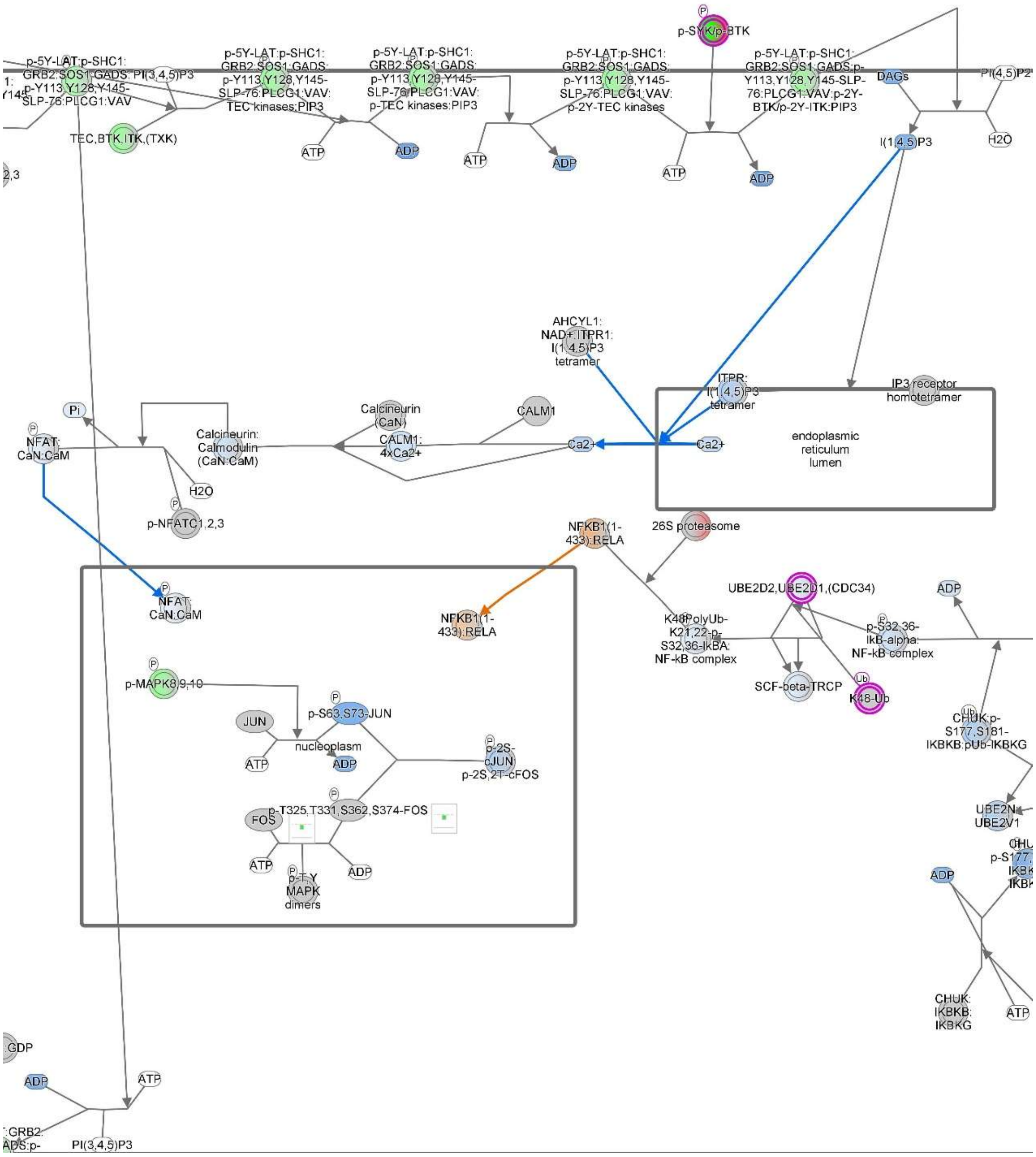
p-SYK/p-BTK
p-5Y-LAT:p-SHC1:GRB2:SOS1:GADS:PI(3,4,5)P3
p-Y113,Y128,Y145-SLP-76:PLCG1:VAV
p-5Y-LAT:p-SHC1:GRB2:SOS1:GADS:p-Y113,Y128,Y145-SLP-76:PLCG1:VAV:TEC kinases:PIP3
p-5Y-LAT:p-SHC1:GRB2:SOS1:GADS:p-Y113,Y128,Y145-SLP-76:PLCG1:VAV:p-TEC kinases:PIP3
p-5Y-LAT:p-SHC1:GRB2:SOS1:GADS:p-Y113,Y128,Y145-SLP-76:PLCG1:VAV:p-2Y-TEC kinases
p-5Y-LAT:p-SHC1:GRB2:SOS1:GADS:p-Y113,Y128,Y145-SLP-76:PLCG1:VAV:p-2Y-BTK/p-2Y-ITK:PIP3
DAGs
PI(4,5)P2
TEC,BTK,ITK,(TXK)
ATP
ADP
I(1,4,5)P3
H2O
AHCYL1:NAD+:ITPR1:I(1,4,5)P3 tetramer
ITPR:I(1,4,5)P3 tetramer
IP3 receptor homotetramer
endoplasmic reticulum lumen
Ca2+
Calcineurin (CaN)
CALM1
CALM1:4xCa2+
Calcineurin:Calmodulin (CaN:CaM)
Pi
NFAT:CaN:CaM
H2O
p-NFATC1,2,3
NFKB1(1-433):RELA
26S proteasome
UBE2D2,UBE2D1,(CDC34)
K48PolyUb-K21,22-p-S32,36-IkBA:NF-kB complex
p-S32,36-IkB-alpha:NF-kB complex
SCF-beta-TRCP
K48-Ub
CHUK:p-S177,S181-IKBKB:pUb-IKBKG
UBE2N:UBE2V1
CHUK:IKBKB:IKBKG
p-MAPK8,9,10
JUN
p-S63,S73-JUN
nucleoplasm
p-2S-cJUN
p-2S,2T-cFOS
FOS
p-T325,T331,S362,S374-FOS
p-T,Y MAPK dimers
GDP
PI(3,4,5)P3

(d)

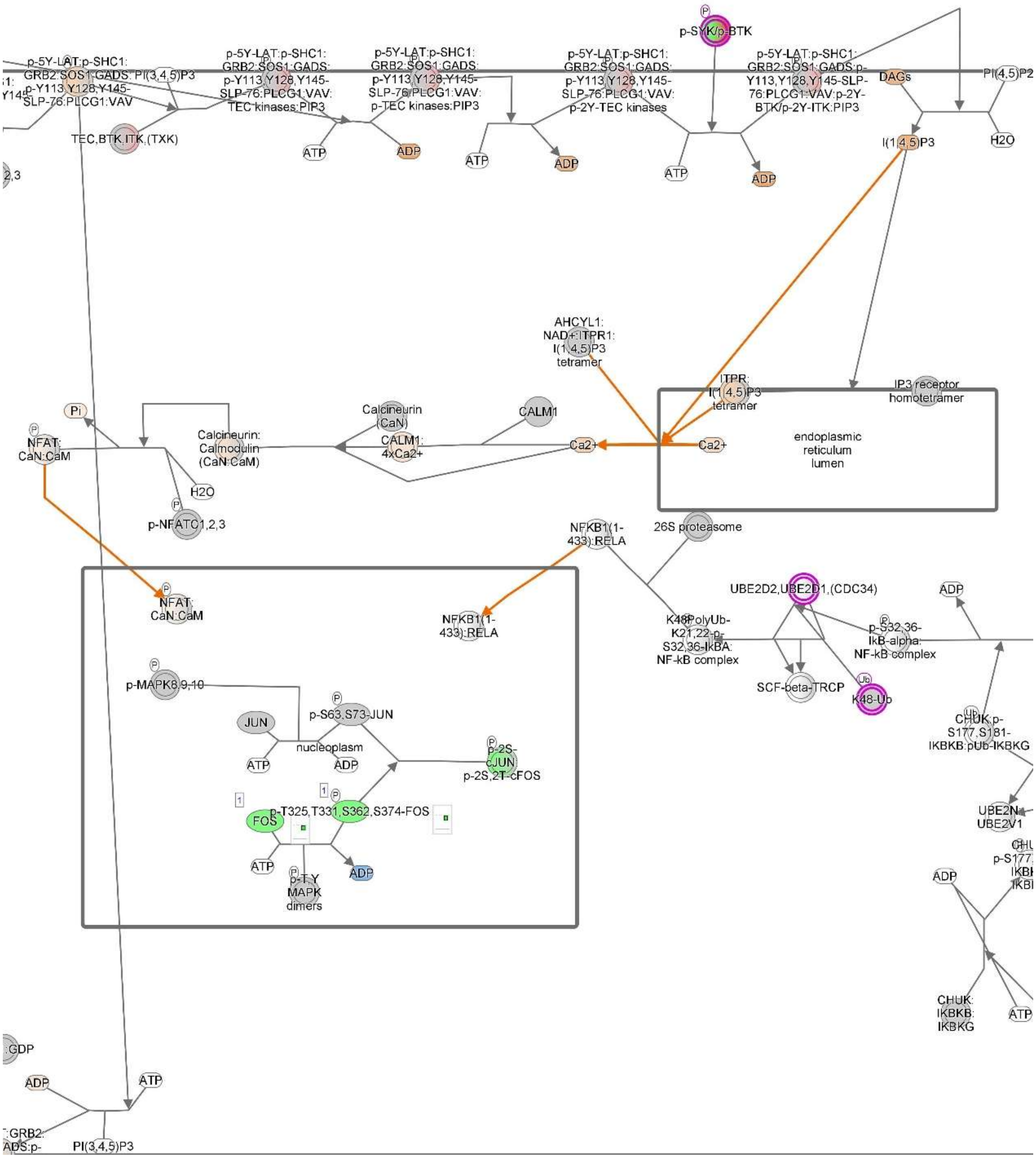

supplement Figure 5. Role of LAT2_NTAL_LAB on calcium mobilization (a) of study dataset (b), non-atopic dataset (c), and high-atopic dataset (d).

(a)

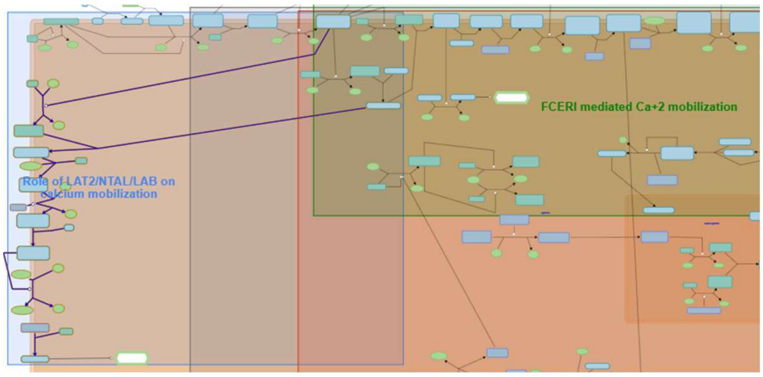

(b)

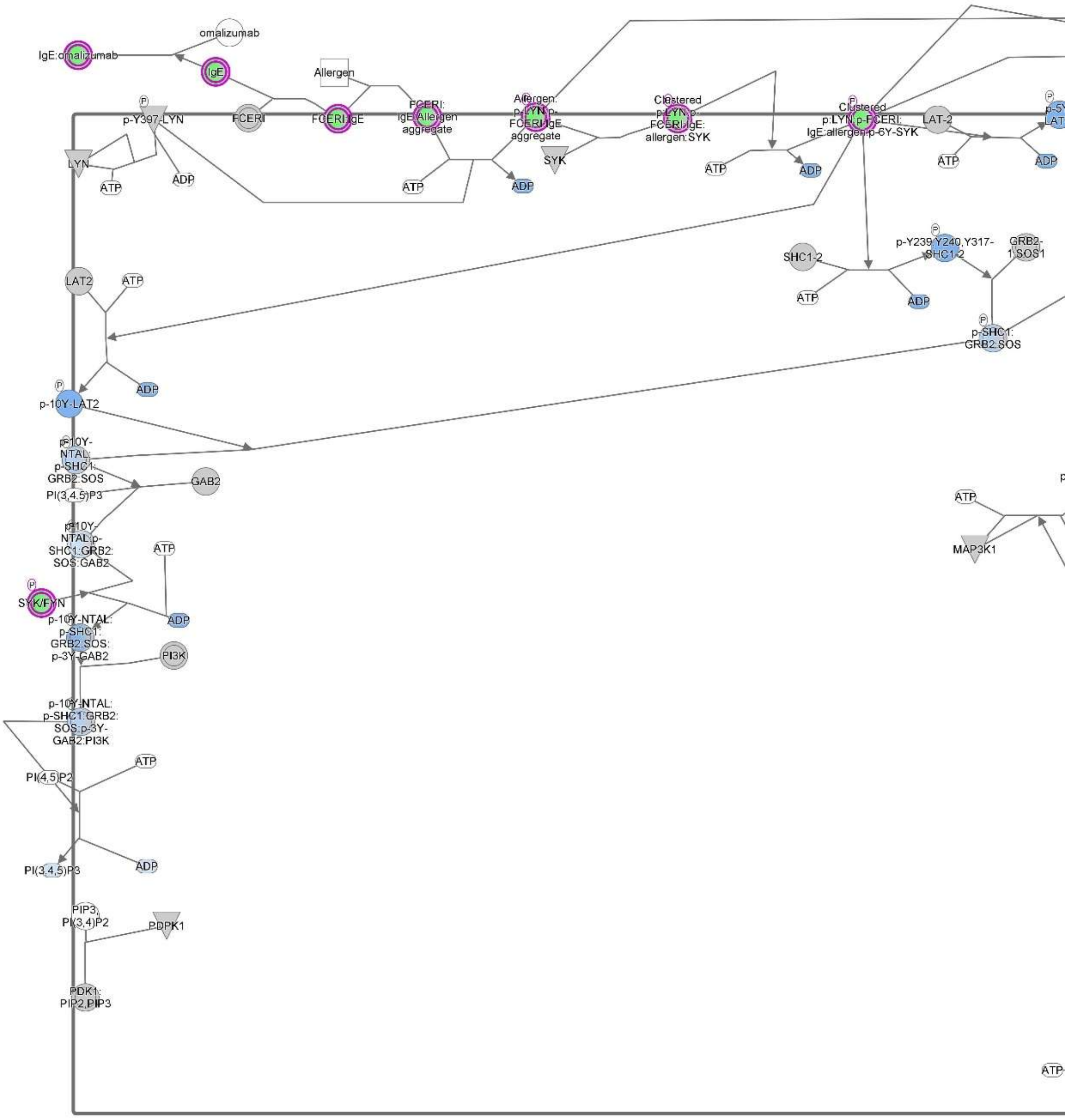

© 2000-2024 QIAGEN. All rights reserved.

(c)

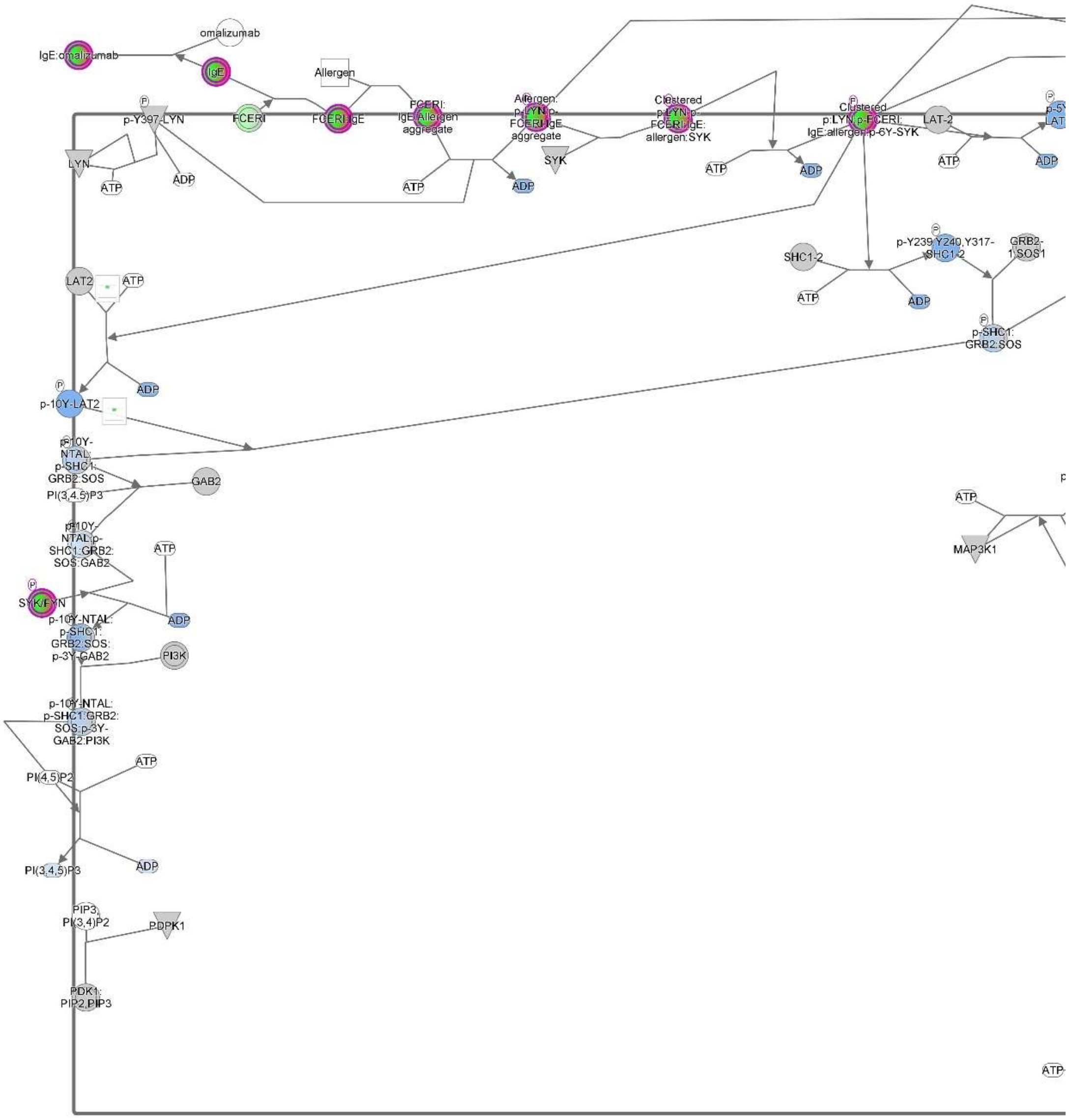

© 2000-2024 QIAGEN. All rights reserved.

(d)

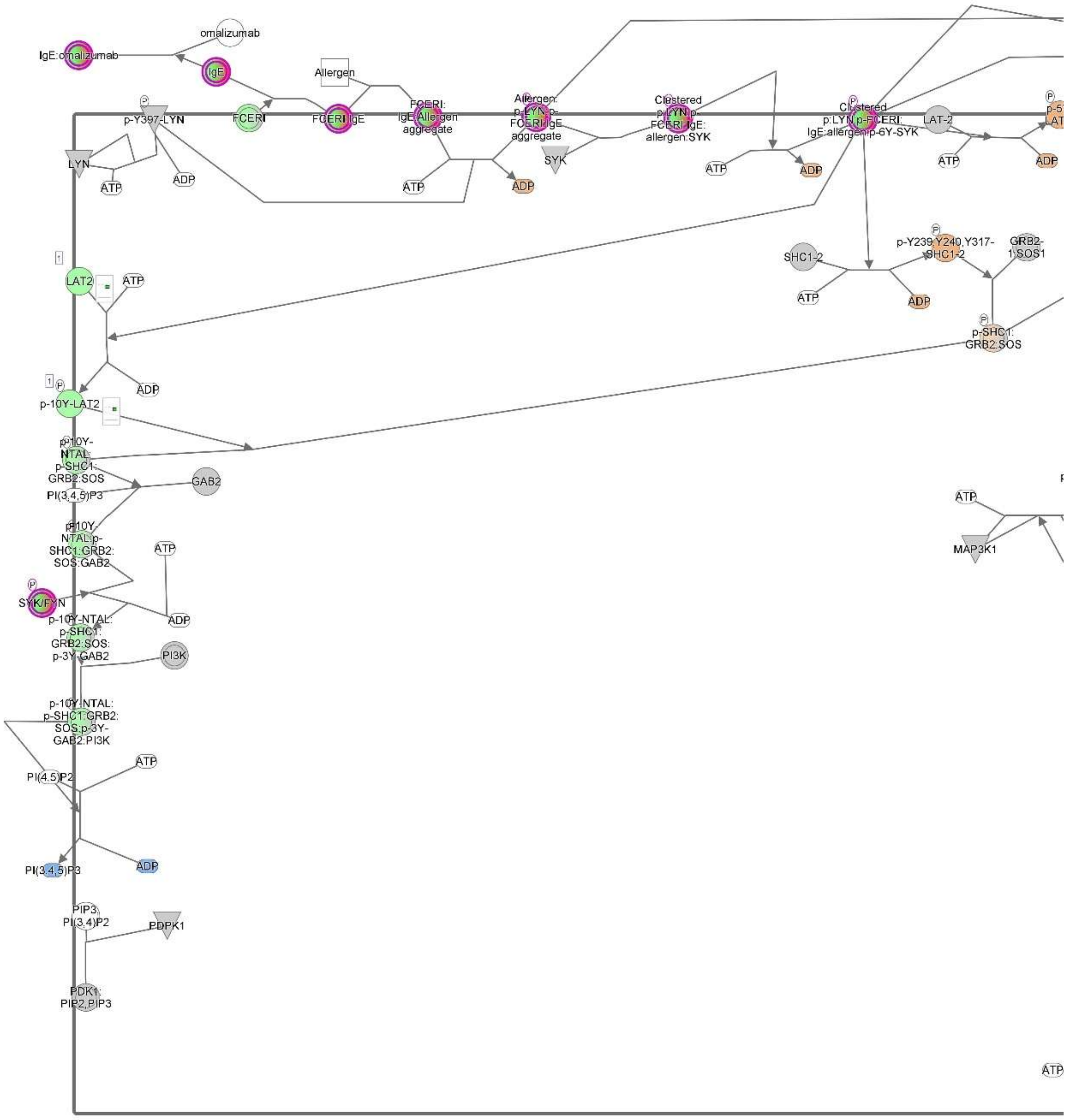

© 2000-2024 QIAGEN. All rights reserved.

SUPPLEMENT TABLES

supplement Table 1. Genes in lightgreen module and their module membership (MM) and gene significance (GS).

Geneid	symbol	description	GS.Eos	p.GS.Eos	MMlightgreen	p.MMlightgreen
ENSG00000228549	RP11-108M9	NA	0.582951	0.001132	0.731459	9.77E-06
ENSG00000162390	ACOT11	acyl-CoA thioesterase 11	0.714307	1.96E-05	0.739691	6.87E-06
ENSG00000184371	CSF1	colony stimulating factor 1	0.500129	0.006724	0.694678	4.10E-05
ENSG00000282608	ADORA3	adenosine A3 receptor	0.479509	0.009824	0.596583	0.000806
ENSG00000224165	DNAJC27-AS1	DNAJC27 antisense RNA 1	-0.44168	0.018619	-0.59002	0.000951
ENSG00000170500	LONRF2	LON peptidase N-terminal domain and ring finger 2	0.569064	0.001577	0.716106	1.83E-05
ENSG00000138395	CDK15	cyclin-dependent kinase 15	0.556335	0.00211	0.752054	3.94E-06
ENSG00000163053	SLC16A14	solute carrier family 16	0.717562	1.72E-05	0.770076	1.65E-06

		member 14				
ENSG00000091181	IL5RA	interleukin 5 receptor subunit alpha	0.752433	3.87E-06	0.966797	6.28E-17
ENSG00000196220	SRGAP3	SLIT-ROBO Rho GTPase activating protein 3	0.813397	1.43E-07	0.864506	3.02E-09
ENSG00000114026	OGG1	8-oxoguanine DNA glycosylase	0.735541	8.22E-06	0.713299	2.04E-05
ENSG00000183625	CCR3	C-C motif chemokine receptor 3	0.49465	0.007454	0.776218	1.21E-06
ENSG00000163606	CD200R1	CD200 receptor 1	0.465421	0.012567	0.639104	0.000251
ENSG00000114315	HES1	hes family bHLH transcription factor 1	0.613409	0.000518	0.862021	3.77E-09
ENSG00000135605	TEC	tec protein tyrosine kinase	0.668115	0.000102	0.760456	2.65E-06
ENSG0000025069	RP11-704M14	NA	0.722719	1.40E-05	0.898801	8.33E-11

6						
ENSG00000213759	UGT2B11	UDP glucuronosyltransferase family 2 member B11	0.748365	4.67E-06	0.893903	1.50E-10
ENSG00000249763	RP11-618I10	NA	0.706633	2.64E-05	0.835123	3.26E-08
ENSG00000135226	UGT2B28	UDP glucuronosyltransferase family 2 member B28	0.690707	4.73E-05	0.872677	1.41E-09
ENSG00000164292	RHOBTB3	Rho related BTB domain containing 3	0.613058	0.000523	0.760436	2.65E-06
ENSG00000113520	IL4	interleukin 4	0.426718	0.02354	0.582199	0.001153
ENSG00000145990	GFOD1	glucose-fructose oxidoreductase domain containing 1	0.513287	0.005215	0.549007	0.002482
ENSG00000237988	OR2I1P	olfactory receptor family 2 subfamily	0.332556	0.083792	0.609197	0.00058

		I member 1 pseudogene				
ENSG00000255587	RAB44	RAB44, member RAS oncogene family	0.765615	2.06E-06	0.837366	2.76E-08
ENSG00000112139	MDGA1	MAM domain containing glycosylphosphatidylinositol anchor 1	-0.48265	0.009286	-0.55262	0.002292
ENSG00000112759	SLC29A1	solute carrier family 29 member 1 (Augustine blood group)	0.74602	5.19E-06	0.949321	1.39E-14
ENSG00000118513	MYB	MYB proto-oncogene, transcription factor	0.695703	3.95E-05	0.787227	6.70E-07
ENSG00000131018	SYNE1	spectrin repeat containing nuclear envelope protein 1	0.659832	0.000133	0.68334	6.13E-05
ENSG0000015372	CNKSR3	CNKSR family	0.57967	0.001226	0.676283	7.80E-05

1		member 3				
ENSG00000071242	RPS6KA2	ribosomal protein S6 kinase A2	0.552205	0.002313	0.755999	3.28E-06
ENSG00000171657	GPR82	G protein-coupled receptor 82	0.669189	9.87E-05	0.850927	9.66E-09
ENSG00000076716	GPC4	glypican 4	0.526818	0.003974	0.552689	0.002289
ENSG00000129675	ARHGEF6	Rac/Cdc42 guanine nucleotide exchange factor 6	0.663386	0.000119	0.808409	1.95E-07
ENSG00000131203	IDO1	indoleamine 2,3-dioxygenase 1	0.719718	1.58E-05	0.881204	6.04E-10
ENSG00000172817	CYP7B1	cytochrome P450 family 7 subfamily B member 1	0.696351	3.86E-05	0.82556	6.40E-08
ENSG00000226393	IFNA20P	interferon, alpha 20, pseudogene	-0.48389	0.00908	-0.64918	0.000186
ENSG00000166016	ABTB2	ankyrin repeat and BTB domain	0.747484	4.86E-06	0.847025	1.32E-08

		containing 2				
ENSG00000183134	PTGDR2	prostaglandin D2 receptor 2	0.687938	5.22E-05	0.928731	1.04E-12
ENSG00000162174	ASRGL1	asparaginase like 1	0.628746	0.000339	0.779417	1.02E-06
ENSG00000168004	HRASLS5	HRAS like suppressor family member 5	0.711512	2.19E-05	0.91171	1.52E-11
ENSG00000175591	P2RY2	purinergic receptor P2Y2	0.694979	4.06E-05	0.779618	1.01E-06
ENSG00000260401	RP11-800A3	NA	0.675104	8.11E-05	0.759308	2.80E-06
ENSG00000119943	PYROXD2	pyridine nucleotide-disulphide oxidoreductase domain 2	0.410262	0.030128	0.608323	0.000594
ENSG00000066468	FGFR2	fibroblast growth factor receptor 2	0.648887	0.000188	0.785874	7.22E-07
ENSG00000050438	SLC4A8	solute carrier family 4 member 8	0.599483	0.000748	0.727292	1.16E-05
ENSG00000135482	ZC3H10	zinc finger CCCH-	0.623257	0.000396	0.609472	0.000576

		type containing 10				
ENSG000000062485	CS	citrate synthase	0.599438	0.000749	0.760851	2.60E-06
ENSG000000185344	ATP6V0A2	ATPase H+ transporting V0 subunit a2	0.602156	0.000698	0.731609	9.71E-06
ENSG000000152207	CYSLTR2	cysteinyl leukotriene receptor 2	0.761229	2.56E-06	0.924109	2.29E-12
ENSG000000151287	TEX30	testis expressed 30	0.482442	0.009321	0.690784	4.72E-05
ENSG000000092067	CEBPE	CCAAT/enhancer binding protein epsilon	0.672945	8.72E-05	0.83604	3.05E-08
ENSG000000092068	SLC7A8	solute carrier family 7 member 8	0.596764	0.000802	0.688976	5.03E-05
ENSG000000100628	ASB2	ankyrin repeat and SOCS box containing 2	0.622912	0.0004	0.788437	6.27E-07
ENSG000000258987	RP11-131H24	NA	0.765198	2.11E-06	0.812818	1.48E-07

ENSG00000140265	ZSCAN29	zinc finger and SCAN domain containing 29	0.754715	3.48E-06	0.783986	8.00E-07
ENSG00000166147	FBN1	fibrillin 1	0.667076	0.000106	0.552972	0.002274
ENSG00000138623	SEMA7A	semaphorin 7A (John Milton Hagen blood group)	0.650562	0.000178	0.849555	1.08E-08
ENSG00000274376	ADAMTS7P1	ADAMTS7 pseudogene 1	0.762313	2.42E-06	0.912238	1.41E-11
ENSG00000103355	PRSS33	protease, serine 33	0.738608	7.20E-06	0.93672	2.32E-13
ENSG00000215148	PRSS41	protease, serine 41	0.764768	2.15E-06	0.937472	1.99E-13
ENSG00000005187	ACSM3	acyl-CoA synthetase medium-chain family member 3	0.734267	8.68E-06	0.814797	1.31E-07
ENSG00000166743	ACSM1	acyl-CoA synthetase medium-	0.763159	2.33E-06	0.800111	3.22E-07

		chain family member 1				
ENSG00000103319	EEF2K	eukaryotic elongation factor 2 kinase	0.76023	2.68E-06	0.900246	6.97E-11
ENSG00000102981	PARD6A	par-6 family cell polarity regulator alpha	-0.51405	0.005138	-0.59615	0.000815
ENSG00000103056	SMPD3	sphingomyelin phosphodiesterase 3	0.78108	9.35E-07	0.959701	7.48E-16
ENSG00000279693	RP11-71L14	NA	0.836001	3.05E-08	0.961036	4.87E-16
ENSG00000182557	SPNS3	SPNS sphingolipid transporter 3 (putative)	0.773133	1.42E-06	0.849821	1.06E-08
ENSG00000161905	ALOX15	arachidonate 15-lipoxygenase	0.714025	1.98E-05	0.93056	7.48E-13
ENSG00000276231	PIK3R6	phosphoinositide-3-kinase regulatory subunit 6	0.80099	3.06E-07	0.948833	1.57E-14

ENSG00000109099	PMP22	peripheral myelin protein 22	0.547671	0.002556	0.79464	4.42E-07
ENSG00000072134	EPN2	epsin 2	0.682455	6.32E-05	0.864568	3.01E-09
ENSG00000167549	CORO6	coronin 6	0.547976	0.002539	0.660143	0.000132
ENSG00000274736	CCL23	C-C motif chemokine ligand 23	0.623698	0.000391	0.81561	1.24E-07
ENSG00000167107	ACSF2	acyl-CoA synthetase family member 2	0.747331	4.89E-06	0.78902	6.07E-07
ENSG00000267452	RP11-1018N14	NA	0.619527	0.000439	0.686041	5.58E-05
ENSG00000134489	HRH4	histamine receptor H4	0.619414	0.00044	0.885704	3.75E-10
ENSG00000174837	ADGRE1	adhesion G protein-coupled receptor E1	0.664723	0.000114	0.829591	4.84E-08
ENSG00000280091	CTC-312O10	NA	0.779026	1.04E-06	0.8846	4.23E-10
ENSG00000268758	ADGRE4P	adhesion G protein-coupled	0.803377	2.65E-07	0.964146	1.68E-16

		receptor E4, pseudogene				
ENSG000010520 5	CLC	Charcot-Leyden crystal galectin	0.595968	0.000818	0.870722	1.70E-09
ENSG000001031 0	GIPR	gastric inhibitory polypeptide receptor	0.67024	9.54E-05	0.732013	9.55E-06
ENSG000010536 6	SIGLEC8	sialic acid binding Ig like lectin 8	0.730869	1.00E-05	0.919506	4.79E-12
ENSG000014240 8	CACNG8	calcium voltage-gated channel auxiliary subunit gamma 8	0.753966	3.61E-06	0.945506	3.49E-14
ENSG000013043 3	CACNG6	calcium voltage-gated channel auxiliary subunit gamma 6	0.635009	0.000283	0.814571	1.32E-07
ENSG000018906 8	VSTM1	V-set and transmembrane domain containing 1	0.693881	4.22E-05	0.756453	3.21E-06
ENSG00	AC00611	NA	0.740223	6.71E-06	0.902644	5.15E-11

000266907	6					
ENSG00000186951	PPARA	peroxisome proliferator activated receptor alpha	0.661896	0.000125	0.753919	3.61E-06
ENSG00000205927	OLIG2	oligodendrocyte lineage transcription factor 2	0.708248	2.48E-05	0.94987	1.21E-14
ENSG00000182240	BACE2	beta-site APP-cleaving enzyme 2	0.588522	0.000987	0.726038	1.22E-05

supplement Table 2. Component molecules of IgE.

Symbol	Synonym(s)	Entrez Gene Name	Location
IGHV1-2	immunoglobulin heavy variable 1-2, V35	immunoglobulin heavy variable 1-2	Other
IGHV1-46	immunoglobulin heavy variable 1-46	immunoglobulin heavy variable 1-46	Other

IGHV1-69	IGHV1-E, immunoglobulin heavy variable 1-69, MYOSIN REACTIVE IMMUNOGLOBULIN HEAVY CHAIN VARIABLE REGION	immunoglobulin heavy variable 1-69	Other
IGHV2-5	immunoglobulin heavy variable 2-5, VH	immunoglobulin heavy variable 2-5	Other
IGHV2-70	immunoglobulin heavy variable 2-70, LOC100996801, VH	immunoglobulin heavy variable 2-70	Extracellular Space
IGHV3-11	immunoglobulin	immunoglobulin	Other

	heavy variable 3-11, VH	heavy variable 3-11	
IGHV3-13	immunoglobulin heavy variable 3-13	immunoglobulin heavy variable 3-13	Extracellular Space
IGHV3-23	DP47, immunoglobulin heavy variable 3-23, V3-23, VH26	immunoglobulin heavy variable 3-23	Extracellular Space
IGHV3-30	immunoglobulin heavy variable 3-30, VH	immunoglobulin heavy variable 3-30	Extracellular Space
IGHV3-33	immunoglobulin heavy variable 3-33, VH	immunoglobulin heavy variable 3-33	Extracellular Space
IGHV3-48	immunoglobulin heavy variable 3-48, VH	immunoglobulin heavy variable 3-48	Other
IGHV3-53	immunoglobulin	immunoglobulin	Other

	heavy variable 3-53, VH	heavy variable 3-53	
IGHV3-7	immunoglobulin heavy variable 3-7, VH	immunoglobulin heavy variable 3-7	Extracellular Space
IGHV4-34	immunoglobulin heavy variable 4-34, VH	immunoglobulin heavy variable 4-34	Other
IGHV4-39	immunoglobulin heavy variable 4-39, VH	immunoglobulin heavy variable 4-39	Other
IGHV4-59	immunoglobulin heavy variable 4-59, LOC100508605, VH	immunoglobulin heavy variable 4-59	Extracellular Space
IGHV7-81	immunoglobulin heavy variable 7-81 (non-	immunoglobulin heavy variable 7-81 (non-	Other

	functional)	functional)	
IGKC	HCAK1, IgG κ, IG KAPPA CONSTANT, Ig kappa light chain, IGKCD, IG LIGHT CHAIN K, IG κ CONSTANT, Ig κ light chain, immunoglobulin kappa constant, immunoglobulin κ constant, Immunoglobulin κ Light Chain, Kappa Light Chain, Km, κ Light Chain	immunoglobulin kappa constant	Extracellular Space
IGKV1-12	immunoglobulin	immunoglobulin	Other

	kappa variable 1-12, immunoglobulin κ variable 1-12, L19	kappa variable 1-12	
IGKV1-16	immunoglobulin kappa variable 1-16, immunoglobulin κ variable 1-16, L1	immunoglobulin kappa variable 1-16	Extracellular Space
IGKV1-17	A30, immunoglobulin kappa variable 1-17, immunoglobulin κ variable 1-17	immunoglobulin kappa variable 1-17	Extracellular Space
IGKV1-33	immunoglobulin kappa variable 1-33, immunoglobulin κ	immunoglobulin kappa variable 1-33	Other

	variable 1-33, O18		
IGKV1-39	immunoglobulin kappa variable 1-39, immunoglobulin κ variable 1-39, O12, O12a	immunoglobulin kappa variable 1-39	Extracellular Space
IGKV1-5	IGKV, immunoglobulin kappa variable 1-5, immunoglobulin κ variable 1-5, L12, L12a, V1	immunoglobulin kappa variable 1-5	Extracellular Space
IGKV1D-12	immunoglobulin kappa variable 1D-12, immunoglobulin κ variable 1D-12, L19	immunoglobulin kappa variable 1D-12	Extracellular Space

IGKV1D-16	immunoglobulin kappa variable 1D-16, immunoglobulin κ variable 1D-16, L15, L15a	immunoglobulin kappa variable 1D-16	Other
IGKV1D-33	immunoglobulin kappa variable 1D-33, immunoglobulin κ variable 1D-33, O8	immunoglobulin kappa variable 1D-33	Extracellular Space
IGKV1D-39	immunoglobulin kappa variable 1D-39, immunoglobulin κ variable 1D-39, O2	immunoglobulin kappa variable 1D-39	Other
IGKV2-28	A19,	immunoglobulin	Other

	immunoglobulin kappa variable 2-28, immunoglobulin κ variable 2-28	kappa variable 2-28	
IGKV2-30	A17, immunoglobulin kappa variable 2-30, immunoglobulin κ variable 2-30	immunoglobulin kappa variable 2-30	Extracellular Space
IGKV2D-28	A3, immunoglobulin kappa variable 2D-28, immunoglobulin κ variable 2D-28	immunoglobulin kappa variable 2D-28	Extracellular Space
IGKV2D-30	A1, immunoglobulin	immunoglobulin kappa variable 2D-	Other

	kappa variable 2D-30, immunoglobulin κ variable 2D-30	30	
IGKV2D-40	immunoglobulin kappa variable 2D-40, immunoglobulin κ variable 2D-40, O1	immunoglobulin kappa variable 2D-40	Other
IGKV3-11	immunoglobulin kappa variable 3-11, immunoglobulin κ variable 3-11, L6, LOC100653210	immunoglobulin kappa variable 3-11	Other
IGKV3-15	immunoglobulin kappa variable 3-15,	immunoglobulin kappa variable 3-15	Extracellular Space

	immunoglobulin κ variable 3-15, L2		
IGKV3-20	13K18, A27, immunoglobulin kappa variable 3-20, immunoglobulin κ variable 3-20	immunoglobulin kappa variable 3-20	Extracellular Space
IGKV3D-20	A11, A11a, immunoglobulin kappa variable 3D-20, immunoglobulin κ variable 3D-20	immunoglobulin kappa variable 3D-20	Extracellular Space
IGKV4-1	B3, Ig kappa chain V-IV region precursor, Ig κ chain V-IV region precursor,	immunoglobulin kappa variable 4-1	Extracellular Space

	immunoglobulin kappa variable 4-1, immunoglobulin κ variable 4-1, LOC101060598		
IGKV5-2	B2, HS.570229, immunoglobulin kappa variable 5-2, immunoglobulin κ variable 5-2	immunoglobulin kappa variable 5-2	Other
IGLC1	IGLC, immunoglobulin lambda constant 1, immunoglobulin λ constant 1	immunoglobulin lambda constant 1	Cytoplasm
IGLC2	IGLC, immunoglobulin lambda constant 2, immunoglobulin λ	immunoglobulin lambda constant 2	Extracellular Space

	constant 2		
IGLC3	IGLC, immunoglobulin lambda constant 3 (Kern-Oz+ marker), immunoglobulin λ constant 3 (Kern-Oz+ marker)	immunoglobulin lambda constant 3 (Kern-Oz+ marker)	Extracellular Space
IGLC7	C7, immunoglobulin lambda constant 7, immunoglobulin λ constant 7	immunoglobulin lambda constant 7	Extracellular Space
IGLV1-36	immunoglobulin lambda variable 1-36, immunoglobulin λ variable 1-36, V1-	immunoglobulin lambda variable 1-36	Other

	11		
IGLV1-40	immunoglobulin lambda variable 1-40, immunoglobulin λ variable 1-40, V1-13	immunoglobulin lambda variable 1-40	Other
IGLV1-44	immunoglobulin lambda variable 1-44, immunoglobulin λ variable 1-44, V1-16	immunoglobulin lambda variable 1-44	Other
IGLV1-47	immunoglobulin lambda variable 1-47, immunoglobulin λ variable 1-47, V1-17	immunoglobulin lambda variable 1-47	Extracellular Space

IGLV1-51	immunoglobulin lambda variable 1-51, immunoglobulin λ variable 1-51, V1-19	immunoglobulin lambda variable 1-51	Extracellular Space
IGLV10-54	immunoglobulin lambda variable 10-54, immunoglobulin λ variable 10-54, V1-20	immunoglobulin lambda variable 10-54	Other
IGLV11-55	immunoglobulin lambda variable 11-55 (non-functional), immunoglobulin λ variable 11-55 (non-functional),	immunoglobulin lambda variable 11-55 (non-functional)	Other

	V4-6		
IGLV2-11	immunoglobulin lambda variable 2-11, immunoglobulin λ variable 2-11, V1-3	immunoglobulin lambda variable 2-11	Extracellular Space
IGLV2-14	immunoglobulin lambda variable 2-14, immunoglobulin λ variable 2-14, V1-4	immunoglobulin lambda variable 2-14	Extracellular Space
IGLV2-18	immunoglobulin lambda variable 2-18, immunoglobulin λ variable 2-18, V1-5	immunoglobulin lambda variable 2-18	Other

IGLV2-23	immunoglobulin lambda variable 2-23, immunoglobulin λ variable 2-23, V1-7	immunoglobulin lambda variable 2-23	Other
IGLV2-33	immunoglobulin lambda variable 2-33 (non-functional), immunoglobulin λ variable 2-33 (non-functional), V1-9	immunoglobulin lambda variable 2-33 (non-functional)	Other
IGLV2-8	immunoglobulin lambda variable 2-8, immunoglobulin λ variable 2-8, V1-2	immunoglobulin lambda variable 2-8	Other
IGLV3-1	immunoglobulin	immunoglobulin	Other

	lambda variable 3-1, immunoglobulin λ variable 3-1, V2-1	lambda variable 3-1	
IGLV3-12	immunoglobulin lambda variable 3-12, immunoglobulin λ variable 3-12, V2-8	immunoglobulin lambda variable 3-12	Other
IGLV3-16	immunoglobulin lambda variable 3-16, immunoglobulin λ variable 3-16, V2-11	immunoglobulin lambda variable 3-16	Other
IGLV3-19	immunoglobulin lambda variable 3-19,	immunoglobulin lambda variable 3-19	Extracellular Space

	immunoglobulin λ variable 3-19, V2-13, VL3L		
IGLV3-21	immunoglobulin lambda variable 3-21, immunoglobulin λ variable 3-21, V2-14	immunoglobulin lambda variable 3-21	Extracellular Space
IGLV3-22	immunoglobulin lambda variable 3-22, immunoglobulin λ variable 3-22, V2-15	immunoglobulin lambda variable 3-22	Other
IGLV3-25	immunoglobulin lambda variable 3-25, immunoglobulin λ	immunoglobulin lambda variable 3-25	Extracellular Space

	variable 3-25, V2-17		
IGLV3-27	immunoglobulin lambda variable 3-27, immunoglobulin λ variable 3-27, V2-19	immunoglobulin lambda variable 3-27	Other
IGLV4-3	immunoglobulin lambda variable 4-3, immunoglobulin λ variable 4-3, V5-1	immunoglobulin lambda variable 4-3	Other
IGLV4-60	immunoglobulin lambda variable 4-60, immunoglobulin λ variable 4-60, V5-4	immunoglobulin lambda variable 4-60	Other

IGLV4-69	immunoglobulin lambda variable 4-69, immunoglobulin λ variable 4-69, V5-6	immunoglobulin lambda variable 4-69	Other
IGLV5-37	immunoglobulin lambda variable 5-37, immunoglobulin λ variable 5-37, V4-1	immunoglobulin lambda variable 5-37	Other
IGLV5-45	immunoglobulin lambda variable 5-45, immunoglobulin λ variable 5-45, V4-2	immunoglobulin lambda variable 5-45	Other
IGLV6-57	immunoglobulin	immunoglobulin	Extracellular

	lambda variable 6-57, immunoglobulin λ variable 6-57, V1-22	lambda variable 6-57	Space
IGLV7-43	immunoglobulin lambda variable 7-43, immunoglobulin λ variable 7-43, V3-2	immunoglobulin lambda variable 7-43	Extracellular Space
IGLV7-46	immunoglobulin lambda variable 7-46, immunoglobulin λ variable 7-46, V3-3	immunoglobulin lambda variable 7-46	Other
IGLV8-61	immunoglobulin lambda variable 8-	immunoglobulin lambda variable 8-	Other

	61, immunoglobulin λ variable 8-61, V3-4	61	

supplement Table 3: Gene symbols associated with CDRs of IgE molecule.

Category	Gene Symbols
Heavy Chain (IGHV)	IGHV1-2, IGHV1-46, IGHV1-69, IGHV2-5, IGHV2-70, IGHV3-11, IGHV3-13, IGHV3-23, IGHV3-30, IGHV3-33, IGHV3-48, IGHV3-53, IGHV3-7, IGHV4-34, IGHV4-39, IGHV4-59
Light Chain Kappa (IGKV)	IGKV1-12, IGKV1-16, IGKV1-17, IGKV1-33, IGKV1-39, IGKV1-5, IGKV1D-12, IGKV1D-16, IGKV1D-33, IGKV1D-39, IGKV2-28, IGKV2-30, IGKV2D-28, IGKV2D-30, IGKV3-11, IGKV3-15, IGKV3-20, IGKV3D-

	20, IGKV4-1, IGKV5-2
Light Chain Lambda (IGLV)	IGLV10-54, IGLV1-36, IGLV1-40, IGLV1-44, IGLV1-47, IGLV1-51, IGLV2-11, IGLV2-14, IGLV2-18, IGLV2-23, IGLV2-8, IGLV3-1, IGLV3-16, IGLV3-19, IGLV3-21, IGLV3-25, IGLV3-27, IGLV4-60, IGLV4-69, IGLV5-37, IGLV5-45, IGLV7-43, IGLV7-46, IGLV8-61
